# Combinatorial in situ cancer vaccines: unlocking broad and enhanced antitumor responses

**DOI:** 10.1038/s41392-026-02821-2

**Published:** 2026-07-23

**Authors:** Weihsuan Chen, Juwon Baig, Hyebeen Choi, Sejin Son

**Affiliations:** 1 Department of Biological Sciences and Bioengineering, Inha University/Industry-Academia Interactive R&E Center for Bioprocess Innovation, Incheon, South Korea; 2 Department of Biological Sciences, Inha University, Incheon, South Korea

**Keywords:** Nanobiotechnology, Vaccines

## Abstract

In situ cancer vaccination, also termed intratumoral immunotherapy, transforms the tumor microenvironment into an endogenous vaccine platform by leveraging the tumor itself as a source of antigens. Unlike conventional tumor-associated antigen (TAA) or personalized neoantigen vaccines that require predefined targets and complex manufacturing, in situ cancer vaccination presents the tumor’s full antigenic repertoire, including TAAs, neoantigens, post-translationally modified epitopes, cryptic peptides, and viral antigens within their native context. This broad antigen exposure elicits robust polyclonal cytotoxic T-cell responses, facilitates epitope spreading, and reduces immune escape driven by tumor heterogeneity. The therapeutic efficacy of this approach arises from the coordinated activation of multiple immune mechanisms. Programmed cell death pathways, including immunogenic apoptosis, pyroptosis, necroptosis, and ferroptosis, release tumor antigens and danger-associated molecular patterns (DAMPs) that promote dendritic-cell activation, efficient cross-presentation, and the priming of durable effector and memory T cells. The incorporation of potent adjuvants and advanced delivery platforms enhances innate-adaptive crosstalk and helps remodel the immunosuppressive tumor microenvironment. Despite these advantages, clinical translation is limited by inconsistent induction of immunogenic cell death, suboptimal intratumoral retention of therapeutics, and barriers to T-cell infiltration. Recent advances in nanomedicine-enabled delivery systems, microenvironmental modulation, and combinatorial strategies, particularly with immune checkpoint blockade, are overcoming these challenges. Collectively, these innovations position in situ cancer vaccination as a patient-tailored, broadly applicable immunotherapy capable of eliciting durable and systemic antitumor immunity.

## Introduction

In situ cancer vaccination, also referred to as intratumoral immunotherapy (ITI), has emerged as a promising immunotherapeutic strategy that converts the tumor microenvironment into a personalized vaccine factory. In contrast to conventional cancer vaccines, which typically involve the exogenous delivery of TAAs or neoantigens in combination with immunoadjuvants, in situ vaccination elicits an immune response directly within the tumor site.^1^ This is typically achieved through the intratumoral injection of immunoadjuvants such as toll-like receptor (TLR) agonists,^2,3^ proinflammatory cytokines,^4^ or oncolytic viruses,^5,6^ which promote local antigen release, uptake, and cross-presentation by antigen-presenting cells (APCs).^7–9^ A defining advantage of in situ vaccination lies in its ability to harness the complete antigenic repertoire of the tumor,^10,11^ which includes both shared TAAs and unique neoantigens generated by tumor-specific mutations. This antigenic diversity enables the generation of a broad and polyclonal cytotoxic T lymphocyte (CTL) response, reducing the risk of antigen-loss variants and enhancing the ability to target heterogeneous tumor subclones.^6,7,10,12,13^ Furthermore, in situ cancer vaccines address major limitations of conventional cancer vaccines, including tumor heterogeneity and antigen-loss variants that can lead to immune escape.^14^ While traditional cancer vaccines, such as peptide-based or dendritic-cell-based vaccines, aim to elicit systemic immunity against predefined antigens, they often suffer from limited immunogenicity and variability in antigen processing. In contrast, in situ vaccination triggers a robust local inflammatory response that not only primes CTLs but also facilitates epitope spreading, thereby enhancing the breadth and durability of the systemic antitumor immune response.^1,15^

Recent clinical investigations have underscored the therapeutic potential of in situ vaccination. In the Phase I/II trial, 27% of patients with indolent lymphoma achieved objective clinical responses at distant, noninjected tumor sites following intratumoral injection of the TLR9 agonist PF-3512676 combined with low-dose radiotherapy, including one complete and three partial responses. Remarkably, several responses were durable, lasting longer than prior therapies and were associated with the induction of tumor-reactive memory CD8⁺ T cells, supporting the systemic efficacy of this in situ vaccination strategy.^14^ More recently, the oncolytic virus talimogene laherparepvec (T-VEC), which induces immunogenic cell death (ICD) and local immune activation, has demonstrated clinical benefit in melanoma, particularly when used in combination with immune checkpoint inhibitors. Collectively, these findings support a strong rationale for integrating in situ vaccination approaches with other immunotherapeutic modalities to enhance clinical efficacy.^16–18^ To complement and extend this principle, additional immunization strategies have been developed to capture a broad spectrum of tumor antigens while addressing the limitations of neoantigen-personalized vaccines. Moreover, their combination with immune adjuvants or immune checkpoint inhibitors may synergistically enhance T-cell priming and improve therapeutic efficacy.

**Fig. 1:**
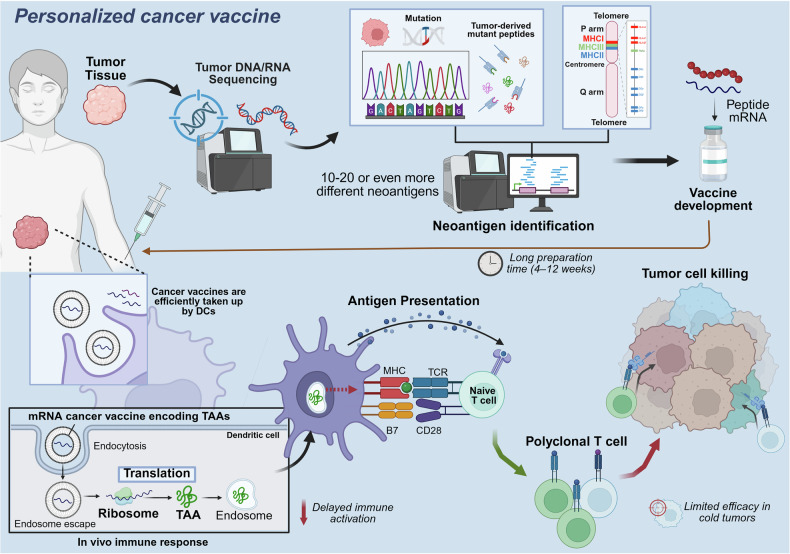
Personalized cancer vaccine. Personalized cancer vaccines are developed based on patient-specific tumor neoantigens identified through genomic sequencing. These preselected antigens are encoded into mRNA or peptide vaccines and delivered to elicit highly specific CD8⁺ T-cell responses. While this strategy enables precision targeting of tumor mutations with minimal off-target effects, it is limited by high production costs, long manufacturing time, and reduced capacity for spontaneous antigen spreading. The figure was generated with BioRender (https://biorender.com)

Traditional cancer vaccines rely on the identification, selection, and ex vivo formulation of a limited set of tumor-specific neoantigens with immunological adjuvants (e.g., peptides, proteins, RNA, DNA).^19–22^ These strategies, while potent against selected targets, are limited by their technical complexity, lengthy manufacturing,^23^ and narrow antigen coverage, leaving them vulnerable to antigen loss and tumor evolution.^24^ (Fig. 1)

**Fig. 2:**
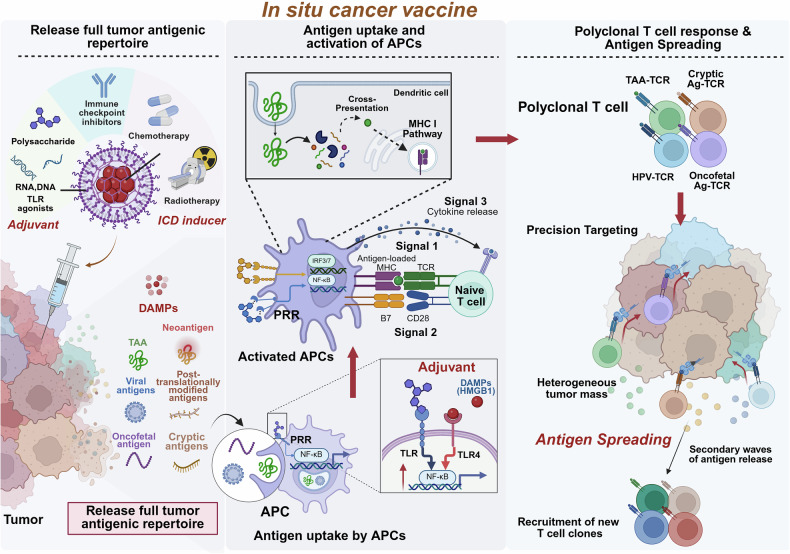
Mechanisms and immunology of in situ cancer vaccines. In situ vaccination involves the intratumoral administration of ICD inducers such as chemotherapy, radiotherapy, or photodynamic therapy combined with immune adjuvants. This strategy promotes the release of the tumor’s full antigenic repertoire. These antigens, together with DAMPs and adjuvants, APCs through PRRs, lead to dendritic-cell maturation and strong T-cell priming via signal 1 (MHC:TCR), signal 2 (CD28:B7), and signal 3 (cytokines). Consequently, in situ cancer vaccines induce a broad polyclonal T-cell response that includes preexisting T-cell boosting and de novo activation of diverse T-cell clones that can address the full spectrum of heterogeneous tumor clones of patients. Furthermore, upon tumor killing, secondary waves of antigen release drive epitope spreading and the activation and recruitment of additional T-cell populations, reinforcing the immune response. The figure was generated with BioRender (https://biorender.com)

Personalized cancer vaccines induce a T-cell response over time, characterized by de novo T-cell activation followed by focused clonal expansion.^25,26^ Due to the limited antigenic diversity of the vaccine, antigen spreading is restricted, resulting in a narrower immune response and reduced recruitment of new T-cell clones targeting secondary tumor antigens.^27,28^ In contrast, in situ cancer vaccines offer a fundamentally different approach by utilizing the tumor itself as a source of antigens and immune activation.^29^ It involves the direct intratumoral administration of immunostimulatory immunoadjuvants such as pattern recognition receptor (PRR) agonists, including toll-like receptor (TLR) agonists, STING agonists, cytokines, or oncolytic viruses, either alone or in combination with ICD inducers, including chemotherapy, radiotherapy, photodynamic therapy (PDT), or nanomaterial-based platforms,^30–32^ thereby enabling rapid, localized immune activation (Fig. 2).

This combinatorial strategy promotes tumor cell death for the release of endogenous cancer antigens, local inflammation, and robust immunological activation for APC activation for T-cell priming via enhanced antigen processing and presentation. A key advantage of in situ cancer vaccines lies in their ability to harness the full antigenic repertoire of the tumor, including shared TAAs, neoantigens generated by somatic mutations, post-translationally modified epitopes, viral antigens (e.g., human papillomavirus (HPV), Epstein–Barr virus (EBV)), and oncofetal proteins. This breadth eliminates the need for antigen selection and enables a rapid, patient-tailored immune response. Following treatment-induced stress or destruction, tumor cells release DAMPs such as calreticulin (CRT),^33^ high mobility group box 1 (HMGB1),^34^ and adenosine triphosphate (ATP),^35^ which are recognized by APCs, especially dendritic cells (DCs), via PRRs,^36^ leading to antigen uptake, maturation, and cross-presentation to T cells. This process initiates the three classical signals of T-cell activation.^37^ These signals collectively lead to the activation and clonal expansion of CD8⁺ CTLs and CD4⁺ helper T cells, along with the generation of long-lived memory T-cell subsets.

Of note, because the tumor’s entire antigenic landscape is presented during in situ cancer vaccination, this strategy elicits a polyclonal T-cell response capable of recognizing and eliminating diverse tumor subclones. Repeated antigen release supports epitope spreading, dynamically expanding the repertoire of activated T-cell clones and enhancing immune surveillance against heterogeneous tumor clones. This process strengthens systemic antitumor immunity and limits immune escape. Specifically, as tumor cells undergo ICD, they release previously unrecognized tumor-associated or neoantigenic epitopes. These antigens are subsequently captured by DCs, processed, and presented via MHC molecules to naive T cells, leading to the activation of new T-cell clones against different tumor epitopes. This progressive diversification of antigen-specific T-cell populations strengthens immune surveillance by enabling the recognition of heterogeneous tumor clones, including those lacking the initially targeted antigens. Therapeutically, the induction of epitope spreading enhances the breadth and adaptability of the antitumor response, helping to mitigate the risk of tumor immune escape driven by antigen loss or clonal evolution. The result is not only a robust primary response but also durable, systemic immunity that supports long-term control of tumor recurrence and metastatic spread. However, this process remains incomplete, as persistent antigenic heterogeneity and T-cell exhaustion can still lead to immune escape. Therefore, the combination of an in situ cancer vaccine with immune checkpoint blockade is necessary to overcome these limitations. These preselected neoantigens are presented by APCs to generate a focused polyclonal T-cell response targeting specific tumor clones. Personalized vaccines provide high precision and potency per epitope but induce delayed and narrower immune responses, with limited epitope spreading and vulnerability to antigen-loss variants. In the initial phase, robust T-cell responses selectively eliminate tumor cells expressing the targeted neoantigens, creating immunologic pressure that shapes tumor evolution. This selective clearance can lead to the transient outgrowth of antigen-loss variants or untargeted clones.

Nonetheless, the inflammatory environment established by personalized neoantigen vaccination promotes the release of additional tumor antigens from dying cancer cells, which are subsequently taken up and presented by APCs. This process recruits new T-cell clones and progressively broadens the overall immune repertoire. Despite this potential, the breadth and kinetics of antigen spreading in personalized vaccination are inherently limited by the initial narrow antigen pool and the time required for successive rounds of tumor elimination and antigen processing. As a result, the immune response may be deep and durable but less adaptable to highly heterogeneous or rapidly evolving tumors compared to in situ cancer vaccination strategies. In contrast, in situ cancer vaccines leverage the entire tumor antigen repertoire from the outset, enabling rapid, broad, and dynamically expanding polyclonal T-cell responses, whereas personalized vaccines deliver delayed but precisely targeted responses against selected neoantigens. Both strategies, despite their different kinetics and breadth, ultimately support the development of durable memory T-cell populations, but in situ cancer vaccines offer greater breadth and adaptability to tumor heterogeneity, whereas personalized vaccines provide enhanced precision and potency. These complementary strengths highlight the potential of integrating in situ cancer vaccines with personalized vaccines for more resilient antitumor immunity.

## Key element of in situ vaccination

### ICD induction

Immunogenic cell death (ICD) represents one of several mechanisms through which in situ vaccination initiates tumor immunity. ICD is a regulated form of cell death that elicits an adaptive immune response by releasing or exposing endogenous immunostimulatory molecules such as calreticulin (CRT), ATP, and HMGB1.^38–42^ Unlike tolerogenic cell death, which is immunologically silent, ICD transforms dying tumor cells into a potent source of tumor-associated antigens and immune-activating signals.^43^ These signals facilitate antigen uptake by DCs, promote DC maturation, and enhance T-cell priming, effectively converting the tumor into an endogenous vaccine.^12,18,38,39^

Apoptotic cell death, the most well-characterized form of regulated cell death, is typically noninflammatory and tolerogenic.^44^ It involves membrane blebbing, chromatin condensation, and caspase activation, culminating in rapid phagocytic clearance of apoptotic bodies without triggering immune activation. The pioneering study by Ronchetti et al. (1999) first demonstrated that apoptotic tumor cells can elicit immune responses, providing an early foundation for the concept of ICD. While apoptosis plays a critical role in maintaining tissue homeostasis, its immunological silencing can limit the activation of antitumor immunity. Moreover, tumor cells often develop resistance to apoptosis through the upregulation of antiapoptotic proteins (e.g., B-cell lymphoma 2 (Bcl-2), survivin) or mutations in proapoptotic regulators (e.g., p53), enabling them to evade both therapy-induced cell death and immune recognition.

In contrast, nonapoptotic forms of regulated cell death, including necroptosis, pyroptosis, and ferroptosis, are often highly immunogenic due to their ability to disrupt plasma membrane integrity, resulting in the release of DAMPs.^45^ These forms of lytic cell death not only circumvent the mechanisms of apoptosis resistance commonly found in tumors but also promote robust immune activation, making them attractive strategies for enhancing ICD in cancer therapy.

**Fig. 3:**
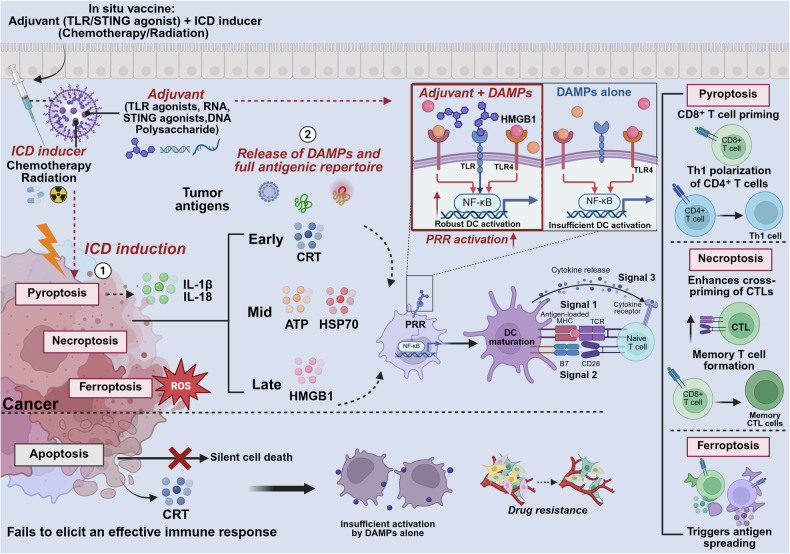
ICD enhanced by in situ cancer vaccination promotes robust DC activation and adaptive immunity. In situ cancer vaccination leverages ICD inducers and immunoadjuvants to initiate ICD, including pyroptosis, necroptosis, and ferroptosis, promoting the release of both DAMPs and a broad spectrum of tumor antigens. These DAMPs engage innate immune receptors, including CD91, P2X7, and TLR4, on DCs, facilitating their maturation and antigen-presenting capacity. Simultaneously, immunoadjuvants delivered by the vaccine formulation activate additional PRRs, such as TLR7/8 or STING, further amplifying DC activation and cytokine release. Effective DC activation drives T-cell priming, facilitating polyclonal CD8⁺ T-cell responses and memory formation. Each ICD exhibits distinct immunological effects: pyroptosis promotes CD8⁺ T-cell priming and Th1 polarization; necroptosis enhances cross-priming and memory CTL generation; and ferroptosis induces antigen spreading, aiding the targeting of heterogeneous tumors. The figure was generated with BioRender (https://biorender.com)

Released DAMPs engage PRRs such as TLRs, NOD-like receptors (NLRs), and the STING (stimulator of interferon genes) pathway in innate immune cells.^41^ This leads to the production of type I interferons and the activation of proinflammatory signaling cascades, driving DC maturation and antigen presentation. As a result, nonapoptotic ICD simultaneously bypasses apoptotic resistance and orchestrates a coordinated innate and adaptive immune response, thereby enhancing T-cell priming and promoting effective tumor clearance and long-term immune surveillance during cancer immunotherapy (Fig. 3).

These DAMPs not only serve as immunostimulatory signals but also ensure the efficient processing and presentation of tumor antigens, making ICD a powerful driver of antitumor immunity and a central component of in situ vaccination efficacy (Table 1).

**Table 1:** Key DAMPs and PAMPs in in situ cancer vaccination and their immunological functions

Molecule	PRR	Function and role
DAMPs (damage-associated molecular patterns)		
CRT	CD91^269^	“Eat-me” signal promoting DC phagocytosis and antigen presentation^270^
Annexin A1	FPR1^271^	Enhances DC phagocytosis and cross presentation^272^
ATP	P2X7^273^	Activates NLRP3 inflammasome, induces IL-1β, and promotes DC maturation^274^
HSP70/HSP90	CD91, TLR2/4^275^	Antigen chaperone dendritic-cell activation NK cell activation^276^
HMGB1	TLR4, RAGE^277^	TLR4 RAGE binding inflammatory signaling antigen presentation^278^
Mitochondrial DNA	cGAS–STING^279^	Oxidative stress induced DNA release cGAS–STING activation I IFN production^280^
PAMPs (pathogen-associated molecular patterns)		
CpG ODN	TLR9	Activates DCs and induces type I IFN and Th1 response
Poly(I:C)	TLR3, MDA5	Triggers antiviral signaling and cytokine production
LPS	TLR4	Induces strong inflammatory activation of DCs/macrophages
Flagellin	TLR5, NLRC4	Induces strong inflammatory activation of DCs/macrophages
STING agonists	STING	Induce type I IFN and enhance antigen presentation
Mannan	TLR4, Dectin-2	Promotes antigen uptake and DC activation

Therefore, understanding how different ICD pathways contribute to the induction of immune responses could provide a mechanistic foundation for effectively harnessing dying tumor cells to enhance therapeutic strategies for in situ cancer vaccination.^38,46^

**Fig. 4:**
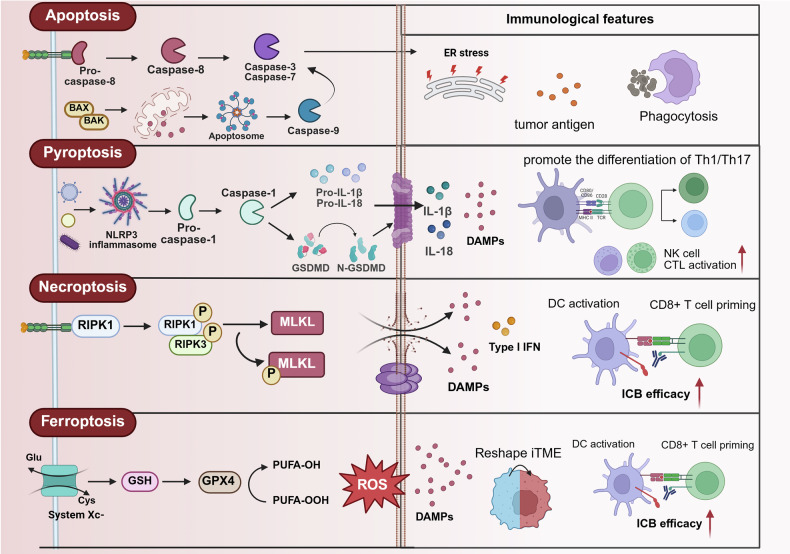
Programmed cell death pathways and their roles in inducing ICD for in situ vaccination enhancement. This illustration depicts four major forms of regulated cell death, apoptosis, pyroptosis, necroptosis, and ferroptosis, that can induce ICD, which is characterized by the release of DAMPs. While apoptosis is generally considered weakly immunogenic under normal conditions, it can acquire ICD-like properties under specific stress conditions, such as ER stress or chemotherapy-induced damage. Apoptosis under such stress facilitates tumor antigen exposure; pyroptosis induces inflammatory cytokines via caspase-1 and GSDMD activation; necroptosis promotes membrane rupture through MLKL oligomerization; and ferroptosis results from ROS-induced lipid peroxidation. The DAMPs released, including CRT, HMGB1, ATP, and IL-1β, activate DCs via PRRs, enhancing antigen presentation, DC maturation, and subsequent CTL responses. These events contribute to the overall efficacy of in situ cancer vaccination. The figure was generated with BioRender (https://biorender.com)

Each PCD modality elicits a distinct repertoire of DAMPs, cytokines, and tumor-derived antigens, which together influence DC activation, antigen cross-presentation, and T-cell priming (Fig. 4).

Apoptosis is typically tolerogenic, although under ER stress, it may expose CRT, promoting phagocytosis by DCs.^33,47^ However, it lacks inflammatory cytokines, often limiting CTL activation and memory.^48^

Necroptosis results in the release of DAMPs, including HMGB1, ATP, and DNA.^49^ These molecules activate the TLR4 and cGAS–STING pathways, promoting type I IFN production and CD8⁺ T-cell recruitment.^50,51^ While necroptosis enhances antigen availability, leukocyte infiltration, and DC cross-presentation, the robust induction of cytokines such as IL-12 and IL-15 generally depends on additional PRR engagement or proinflammatory cues that fully activate DCs via nuclear factor kappa-light-chain-enhancer of activated B cells (NF-κB) and interferon regulatory factor (IRF) signaling pathways.^52–54^ In particular, NF-κB signaling downstream of TLR4 and RIPK1–TAK1 complexes induces the transcription of proinflammatory cytokines (e.g., TNF-α, IL-6, IL-12p40) and costimulatory molecules, whereas IRF3 activation through the cGAS–STING–TBK1 axis promotes type I interferon production, upregulates antigen-processing machinery, and supports IL-15 expression. Together, these pathways link necroptotic DAMP release to innate immune activation, ensuring effective DC licensing and subsequent priming of cytotoxic T-cell responses.

Agents such as GSK3145095, also known as compound 6, are highly selective, orally bioavailable small‑molecule, receptor-interacting serine/threonine-protein kinase 1 (RIPK1) inhibitors with potent activity in blocking RIP1 kinase-dependent cellular pathways. Preclinical evaluation showed that GSK3145095 not only suppresses RIP1‑mediated necroptotic signaling but also promotes a tumor suppressive T-cell phenotype in ex vivo pancreatic cancer cultures. Compound 6 is currently under Phase I clinical investigation for pancreatic adenocarcinoma and other selected solid tumors.^55^

Pyroptosis leads to pore formation and the release of ATP, HMGB1, IL-1β, and IL-18. These promote DC recruitment, costimulatory molecule expression, and antigen presentation.^56–58^ IL-1β drives Th17 differentiation; IL-18 and IL-12 induce IFN-γ and Th1 polarization.^59^ These cytokines enhance CD8⁺ T and NK cell function.^58,60,61^

Pyroptosis can turn “cold” tumors “hot” and boost immune checkpoint therapy. Gasdermin-based nanoparticles,^62^ ultrasound-mediated pyroptosis induction,^63^ and combination with STING agonists have demonstrated synergistic therapeutic outcomes.^64^ By coupling immunogenic antigen release with intrinsic adjuvanticity, pyroptosis serves as a powerful effector mechanism to potentiate in situ vaccination efficacy.

Ferroptosis releases MDA and 4-HNE,^65–67^ which activate DCs and enhance cross-presentation.^68^ It depletes M2-TAMs,^69,70^ disrupts TGF-β signaling,^71^ and promotes chemokines such as CXCL1,^72^ leading to increased CD8⁺ and NK cell infiltration and reduced Tregs and MDSCs.^73,74^ Combined with ICB or innate agonists, ferroptosis improves effector responses in “cold” or apoptosis-resistant tumors.^75^ While PCD pathways support ICD, their clinical translation depends on precise delivery, controlled activation, and immune coordination.^76^

### Immunoadjuvants: robust activation of antigen-presenting cells

Conventional vaccine adjuvants are simple, well-established substances such as mineral salts, emulsions, and select microbial or plant extracts. The most widely used group is aluminum-based salts (alum), including aluminum hydroxide and aluminum phosphate.^77^ Commercial vaccines with alum include diphtheria, tetanus, and pertussis vaccines, hepatitis A and B vaccines, and several pediatric vaccines. Oil-in-water emulsions such as MF59 (in Fluad) and AS03 (in Pandemrix) use squalene oil with surfactants and prolong antigen release.^78^ Water-in-oil products include Montanide ISA series, widely used in veterinary and experimental vaccines.^79^ Freund’s adjuvant, containing mineral oil and inactivated Mycobacterium, is restricted to preclinical research due to its high toxicity. Plant saponins, notably QS-21, are used in advanced adjuvant systems such as AS01 for Shingrix.^80^ These adjuvants primarily act by depot formation and general immune activation, not directing immune profiles. This contrasts with modern adjuvants designed for precise and targeted immunological modulation. In the conventional framework, immunoadjuvants are defined as molecules or compounds possessing intrinsic immunomodulatory properties that, when coadministered with an antigen, significantly amplify antigen-specific immune responses compared to antigen administration alone.^81,82^ Traditionally, their role in vaccine design was primarily centered on enhancing the magnitude of the immune response, without precise control over its qualitative attributes. These adjuvants broadly stimulated immunity, often through generalized activation of innate immune pathways, and improved overall immunogenicity regardless of the functional profile.^22^ While they could influence parameters such as T helper cell bias (Th1 vs. Th2), CD8⁺ cytotoxic versus CD4⁺ helper T-cell ratios, or antibody isotype distribution, such effects were typically incidental rather than deliberately engineered.^83,84^

Modern immunoadjuvants are rationally designed agents that elicit highly specific and potent immune responses, surpassing the broad, generalized effects of traditional adjuvants. These adjuvants not only accelerate and amplify immune activation but also promote robust, long-lasting immunological memory, especially T-cell memory, which is critical for durable protection against complex pathogens and cancers.^83,85^ They expand the breadth, specificity, and functional affinity of antibody and T-cell responses, enabling vaccines to combat a wide range of disease targets. Mechanistically, modern immunoadjuvants often act as PAMP or DAMP mimetics, targeting pattern recognition receptors (PRRs) on APCs to initiate precise innate immune signaling cascades.^86^ APC activation for adaptive immunity relies on three signals: antigen presentation (signal 1), upregulation of costimulatory molecules (signal 2), and cytokine-mediated T-cell polarization (signal 3).^87^ While signal 1 alone can lead to tolerance, coordinated signals 1 and 2 are required for T-cell activation, and all three are necessary for a fully functional response. Immunoadjuvants specifically drive signals 2 and 3 by activating PRRs and downstream transcription factors such as NF-κB and IRFs,^88^ resulting in cytokine production (IL-6, IL-12, TNF-α, type I interferons) and increased costimulatory marker expression.^89^ This molecular orchestration promotes complete dendritic-cell maturation and the efficient priming of diverse antigen-specific T-cell subsets, including Th1, Th2, Th17, and cytotoxic CD8⁺ T cells.^90^ Altogether, modern immunoadjuvants function as precise accelerators and directors, enabling next-generation vaccines to induce durable, rapid, and targeted protective immunity.

In the context of in situ cancer vaccines, immunoadjuvants play a central role in orchestrating potent T-cell-mediated antitumor immunity. Upon direct administration into the tumor site, modern immunoadjuvants activate PRRs on DCs and other APCs.^37^ This activation drives DC maturation, upregulation of costimulatory molecules (CD80, CD86, CD40), and secretion of proinflammatory cytokines (e.g., IL-12, TNF-α, type I interferons),^83^ which are essential for effective processing and presentation of tumor-associated antigens on MHC molecules.^91^ Such enhanced antigen presentation enables the priming and clonal expansion of tumor-specific CD8⁺ cytotoxic T lymphocytes. These CTLs infiltrate the immunosuppressive tumor microenvironment (TME), recognize cancer cells via MHC-I-embedded tumor antigen peptides, and induce apoptosis through perforin/granzyme pathways.^92^ Immunoadjuvants also facilitate Th1 differentiation,^37^ support CTL functionality and persistence, and induce the generation of long-lived memory T cells critical for durable tumor control.^93,94^ This results not only in local tumor regression but also in systemic antitumor responses, sometimes leading to the abscopal effect (targeting distant, noninjected lesions).

Despite these benefits, the efficacy of immunoadjuvant monotherapy is fundamentally limited by the immunogenicity of endogenous tumor antigens.^95^ Many tumor cells do not undergo sufficiently immunogenic cell death, resulting in weak or inadequate antigenic and danger signals that fail to initiate robust, long-lasting adaptive responses, especially within highly immunosuppressive TMEs.^96^ The immunosuppressive features of the TME often blunt T-cell effector functions, induce exhaustion, and dampen cytotoxic activity. Immunoadjuvant-driven activation must therefore be robust enough to overcome these local suppressive signals, promoting sustained effector differentiation and preventing T-cell exhaustion. In summary, the integration of immunoadjuvant mechanisms systematically ignites the “cold” TME, transforming it into an immunologically active site capable of recruiting, activating, and sustaining antitumor T-cell responses. This shortcoming frequently translates to incomplete tumor rejection, limited formation of immune memory, and insufficient systemic (abscopal) effects, thereby restricting the clinical utility of immunoadjuvant-only approaches.

These insights provide a compelling rationale for combining immunoadjuvants with ICD inducers, which enhance the release of TAAs and danger signals to more effectively prime and sustain T-cell-mediated antitumor immunity. Combining immunoadjuvants with ICD inducers overcomes these obstacles by ensuring that abundant tumor-associated antigens and DAMPs are released, further enhancing DC activation and antigen presentation. This synergistic strategy generates a more profound and durable antitumor immune response, establishes potent memory T-cell populations, and significantly increases the likelihood of effective tumor eradication and long-term protection.

While ICD inducers such as doxorubicin, oxaliplatin, or photodynamic therapy release DAMPs, including CRT, ATP, and HMGB1, the resulting APC activation is often incomplete due to immunosuppressive cues within the tumor microenvironment, such as IL-10, TGF-β, or PGE₂,^97,98^ that limit costimulatory signaling and antigen presentation.^99^ Therefore, potent immunoadjuvants are required to overcome these barriers by reinforcing DC activation, enhancing antigen uptake, and sustaining cross-presentation of ICD-derived tumor antigens.

In situ cancer vaccines integrate ICD inducers with PRR-targeting adjuvants to create a “danger-signal-rich” tumor milieu, where endogenous DAMPs and exogenous PAMPs costimulate innate sensors. This dual activation establishes spatially and temporally coordinated DC maturation, CCR7-mediated migration to tumor-draining lymph nodes, and polyclonal CD8⁺ and CD4⁺ T-cell priming. Mannan-based nanocapsules exemplify such dual-acting platforms by coengaging Dectin-2 and TLR4, enhancing Th17-polarizing cytokine responses and CD40 upregulation.^100^

In the study by Lindsay M. Scheetz et al., synthetic high-density lipoprotein (sHDL) nanoparticles encapsulating docetaxel (DTX) were combined with the TLR9 agonist CpG to enhance the efficacy of chemotherapy-induced ICD.^101^ The sHDL nanocarrier efficiently delivered DTX to MC38 colon adenocarcinoma cells through SR-B1-mediated uptake, and the addition of CpG significantly amplified the antitumor effects. Among the seven mice treated with DTX-sHDL/CpG, two exhibited complete tumor regression, and no systemic toxicity was observed in any of the animals throughout the treatment course. This study clearly demonstrated the superior antitumor efficacy of codelivering docetaxel and CpG on sHDL nanoparticles compared with docetaxel monotherapy in colon carcinoma, highlighting how the combination of a chemotherapeutic ICD inducer with a PRR agonist can synergistically strengthen innate activation and promote durable antitumor immunity.

In addition to DCs and macrophages, B cells also represent an important component of the adaptive immune activation triggered by in situ cancer vaccines. Memory B cells and their antibodies constitute a central component of immune memory, enabling sustained antigen recognition even in the absence of continuous stimulation.^102,103^ However, the precise role of B cells in cancer immunotherapy responses remains incompletely understood. Emerging evidence indicates that beyond antibody production, B cells can enhance antitumor immunity by presenting antigens and providing costimulatory signals to T cells.^104,105^ Although the majority of in situ cancer vaccine research focuses on T-cell-mediated antitumor effects, mounting evidence suggests that humoral immunity mediated by B cells and antibodies may also contribute meaningfully to antitumor responses.^106,107^ Preclinical studies have shown that potent in situ cancer vaccine regimens, such as those combining radiotherapy with intratumoral immunocytokines (e.g., hu14.18‑IL2) and immune checkpoint blockade (e.g., anti‑CTLA‑4), can induce endogenous tumor-specific IgG production and generate humoral immune memory not observed following surgical resection alone.^108^ In murine B78 melanoma models, this combinatorial in situ cancer vaccine triggered robust activation of B cells, leading to class-switched IgG responses (IgG1, IgG2b, IgG2c, and IgG3) that specifically recognized tumor antigens shared between B78 and B16 cells. Serum antibody titers rose within three weeks of treatment and were further boosted upon tumor rechallenge, indicating the establishment of memory B-cell responses. This indicates that B cells can be activated in a T-cell-dependent manner by an in situ cancer vaccine, potentially contributing to antibody-dependent cellular cytotoxicity, antigen uptake by APCs, or long-term immune surveillance.^108,109^

Beyond canonical protein or peptide antigens, B cells can also recognize glycan-dependent epitopes on aberrantly glycosylated tumor surface proteins. MUC1 glycopeptide vaccines, composed of synthetic MUC1 tandem repeats with tumor-specific glycosylation, are being evaluated in clinical trials for breast, ovarian, pancreatic, and other cancers.^110–113^ These vaccines aim to elicit a dual immune response, combining antibody-mediated recognition of glycan-dependent epitopes with cytotoxic T-cell responses against peptide regions presented by MHC molecules. Moreover, newly developed vaccine formulations have adopted MUC1 glycopeptides as B-cell epitopes,^114^ coupled with heterologous T-cell epitopes or Toll-like receptor (TLR) agonists, to synergistically enhance both humoral and cellular antitumor immunity.^115^ Overall, these insights reposition B cells from passive antibody producers to active participants in in situ cancer vaccine-induced immunity, where their recognition of glycan-dependent epitopes and support of T-cell responses collectively broaden the scope and durability of antitumor immunity.

### Harnessing the full tumor antigen diversity for T-cell activation

The effectiveness of cancer immunotherapy fundamentally relies on the immune system’s ability to recognize and eliminate tumor antigens. However, tumor heterogeneity and immune pressure often drive antigen loss or clonal evolution, limiting the effectiveness of approaches that rely on a narrow antigen pool.

Unlike conventional cancer vaccines that require the prior identification and formulation of specific TAAs, in situ cancer vaccines lead to the in situ release of the tumor’s full antigenic repertoire in its native and immunologically relevant context.^1^ While TAAs have been investigated, their clinical utility is limited by immune tolerance. In contrast, tumor-specific neoantigens, post-translationally modified epitopes, oncofetal proteins, and cryptic peptides (Table 2) represent complementary targets, providing a broader and more unbiased antigen landscape for immune recognition. The induction of polyclonal T-cell responses against this diverse antigen pool not only enhances tumor clearance but also helps mitigate immune escape driven by antigen loss or clonal evolution. Furthermore, combining in situ vaccination with complementary strategies such as immune checkpoint blockade to prevent T-cell exhaustion or repeated intratumoral dosing to sustain antigen exposure can further strengthen immune surveillance and improve long-term tumor control.

**Table 2:** Tumor antigen classes comprising the full antigenic repertoire in situ

Antigen class	Source/origin	Immunogenicity	Examples	Clinical relevance
TAAs	Self-derived proteins overexpressed in malignant cells	Low to moderate	HER2/neu, CEA	Improved by checkpoint inhibitors
Tumor-specific neoantigens	Somatic mutations, gene fusions, aberrant splicing	High	KRAS G12D, fusion peptides, retained introns	Basis for personalized vaccines
Post-translationally modified antigens	Abnormal glycosylation, phosphorylation, citrullination, etc.	Moderate to high	MUC1 (Tn, STn, TF), citrullinated peptides	Novel epitopes; glycopeptide vaccines in trials
Oncofetal antigens	Embryonic/developmental proteins re-expressed in tumors	Moderate	NY-ESO-1, AFP	Tumor-selective; targets of peptide/TCR-based therapies
Viral antigens	proteins integrated into the host genome	High	HPV E6/E7, EBV LMP1	Nonself antigens; validated vaccine targets in HPV/EBV cancers
Cryptic/noncanonical peptides	Translation from cryptic ORFs, retained introns, or UTR regions	Variable	Unannotated ORF-derived peptides	Emerging targets; identified via advanced sequencing

**Fig. 5:**
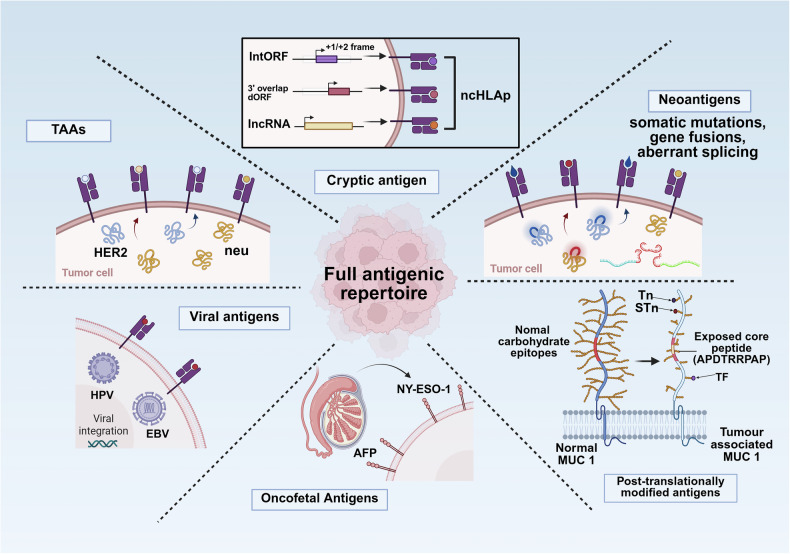
In situ cancer vaccination induces the release of a full tumor antigenic repertoire. Local tumor destruction leads to the release of a diverse array of tumor-derived antigens into the tumor microenvironment, including TAAs (e.g., HER2, neu), tumor-specific neoantigens (from mutations, gene fusions, or splicing events), post-translationally modified antigens (e.g., altered MUC1 glycoforms), oncofetal antigens (e.g., NY-ESO-1, AFP), viral antigens (e.g., HPV, EBV), and cryptic antigens. Together, these constitute the full antigenic repertoire available for uptake and cross-presentation by APCs. The figure was generated with BioRender (https://biorender.com)

The full antigenic repertoire released through in situ cancer vaccination encompasses a diverse array of tumor-associated antigen classes (Fig. 5), each exhibiting distinct immunological characteristics and therapeutic implications.^116^

Classical TAAs, such as carcinoembryonic antigen (CEA) and human epidermal growth factor receptor 2/neural (HER2/neu),^117^ are self-derived proteins aberrantly overexpressed in malignant cells. Although these antigens can be immunogenic, their origin from self-tissues subjects them to central and peripheral tolerance,^118^ which often necessitates the concurrent use of immune checkpoint inhibitors such as antibodies against programmed cell death protein 1 (anti-PD-1) antibodies to achieve effective cytotoxic T-cell activation.^119^

In contrast, tumor-specific neoantigens arise from somatic mutations, gene fusions, or aberrant splicing events^120,121^ and are absent from normal tissues, making them highly immunogenic. Because they are entirely novel to the host immune system, neoantigens are recognized as nonself and can elicit robust CTL responses without being subject to central or peripheral immune tolerance.^122,123^ Unlike TAAs, which are often self-proteins overexpressed in tumors and present in normal or immune-privileged tissues, neoantigens are tumor-exclusive and offer a higher degree of specificity and safety as immunotherapy targets. The immunological advantages of neoantigens make them ideal candidates for personalized cancer vaccines.^32^ For example, the combination of the personalized neoantigen vaccine NEO-PV-01 with a PD-1 inhibitor has been used to treat patients with advanced melanoma, non-small-cell lung cancer, or bladder cancer.^124^ Clinical trial NCT03953235 evaluated SLATEv1, an off-the-shelf shared neoantigen vaccine targeting common driver mutations such as KRAS and TP53. Although the combination was well tolerated and induced neoantigen-specific CD8⁺ T-cell responses, it showed limited clinical efficacy, with a 0% objective response rate and a median progression-free survival of 1.9 months.^123,125^

Additionally, tumors frequently undergo post-translational modifications such as aberrant glycosylation,^115,126^ phosphorylation, citrullination, or acetylation. These alterations can create neoepitopes that distinguish tumor antigens from their normal counterparts and can be selectively targeted by vaccines. A well-characterized example is abnormally glycosylated mucin-1 (MUC1), a mucin protein that, in tumors, carries truncated O-linked glycans such as Tn (GalNAcα1-O-Ser/Thr) and sialyl-Tn (STn). These tumor-associated glycoforms expose both peptide backbones and glycan-dependent neoepitopes not present in normal tissues. Glycan-dependent epitopes are primarily recognized by B cells and antibodies, as their antigenicity arises from the three-dimensional configuration of tumor-specific carbohydrate structures. However, aberrant glycosylation can also modify antigen processing and MHC loading, giving rise to glycopeptide neoantigens that are capable of engaging T-cell receptors (TCRs) in a distinct manner.^115,126^ On the other hand, several studies support the potential of targeting glycosylation in cancer immunotherapy. For instance, targeting PD-L1 glycoforms has emerged as a promising strategy to improve immune checkpoint therapy, and recent efforts have aimed to incorporate glycan epitopes on PD-L1 into biomarker panels for anti–PD-L1/PD-1 treatment stratification.^127^

Another important category includes oncofetal antigens,^128^ including alpha-fetoprotein (AFP) and New York esophageal squamous cell carcinoma 1 (NY-ESO-1).^129,130^ These proteins are typically expressed during embryogenesis but become re-expressed in cancers while remaining largely silent in adult tissues, making them ideal targets for TCR-based and peptide-based immunotherapies,^129,131,132^

Cryptic and noncanonical antigens constitute a class of tumor-associated peptides that arise from nontraditional genomic and transcriptomic regions,^133–136^ Recent immunopeptidomic and translatome profiling studies have confirmed that cryptic peptides are endogenously processed and presented on MHC class I molecules across a variety of malignancies, including glioblastoma, melanoma, acute myeloid leukemia (AML), and pancreatic ductal adenocarcinoma (PDAC). In AML, Cai et al. identified over 10,000 noncanonical MHC-associated peptides (ncMAPs), many derived from noncoding regions such as lncRNAs and UTRs, several of which showed strong immunogenicity.^137^ Likewise, in atypical teratoid/rhabdoid tumors (AT/RTs), a significant fraction of the MHC class I immunopeptidome is composed of cryptic peptides derived from unannotated ORFs and alternative reading frames.^138^

In addition to self- and mutation-derived tumor antigens, oncogenic virus-encoded antigens provide another source of nonself, highly immunogenic targets. Viruses such as human papillomavirus (HPV) and Epstein–Barr virus (EBV) are causally associated with the development of several cancers.^139^ Therapeutic HPV vaccines, especially those targeting E6 and E7 oncoproteins from high-risk subtypes (e.g., HPV16/18), have been shown to activate CD8⁺ T cells and induce targeted cytotoxicity against infected cancer cells. These promising results suggest that viral antigens can be harnessed as part of the antigenic repertoire exploited in in situ cancer vaccine peptides, and post-translationally modified antigens that are released and processed in their native immunological context during immunogenic cell death.^21^ This enables the de novo priming of T cells against a wide array of tumor antigens, many of which may be unrecognized by conventional approaches. The result is the induction of a diversified and polyclonal CD8⁺ T-cell response, supported by epitope spreading and the formation of multiple memory T-cell subsets, including central memory (TCM), effector memory (TEM), and tissue-resident memory (TRM) cells, that sustain long-term immunological surveillance and facilitate durable tumor control.^140^

## Limitations of in situ cancer vaccination

Despite their many advantages, in situ cancer vaccines face several key limitations that continue to impede clinical translation. A central challenge lies in the inconsistency of ICD induction. Although chemotherapeutic agents, radiotherapy, and oncolytic viruses have been shown to promote DAMP release,^141^ the magnitude and duration of these signals vary across tumor types and treatment contexts, often falling short of eliciting sufficient DC maturation and T-cell priming.^41,141^ In cases where DAMP emission is partial or transient, tumor cell death may be immunologically silent or even tolerogenic, failing to initiate effective adaptive immunity.^142^ Additionally, the immunosuppressive TME poses a formidable barrier to in situ cancer vaccine efficacy. Characterized by abundant regulatory T cells (Tregs), myeloid-derived suppressor cells (MDSCs), and M2-polarized tumor-associated macrophages (TAMs), all of which inhibit antigen presentation and effector T-cell function.^143–145^ Physical barriers, such as dense stromal matrix and aberrant vasculature, further impede immune cell infiltration and intratumoral drug distribution.^146^

In situ cancer vaccines often fail to generate robust and durable immunological memory. In immunologically “cold” tumors lacking sufficient innate activation, type I IFN signaling, and conventional type-1 dendritic-cell (cDC1) recruitment, the local immune response fails to propagate systemically or generate long-lived memory T cells.^147,148^ These limitations underscore the need for rational combination therapies to enhance ICD potency, remodel the TME, and support effective memory T-cell development.

### Limited ICD induction

A central challenge in in situ cancer vaccination is ensuring that tumor cells not only undergo death but also lead to the immunologically productive release of TAAs and DAMPs. Effective antitumor immunity requires that TAAs and DAMPs be released in a context that activates innate immune sensors, licenses APCs, and promotes robust CTL responses. However, simply inducing tumor cell death is insufficient, and antigen exposure must be coordinated with innate immune activation to drive durable immunity.

Conventional ICD inducers, such as doxorubicin, oxaliplatin, and bortezomib,^149^ have demonstrated the ability to promote DAMP and TAA release.^150^ However, several delivery-related factors hinder their immunogenic efficacy in vivo.^39^ Many of these agents require careful formulation due to limited solubility and stability, and they often lack tumor specificity, leading to systemic distribution and suboptimal accumulation at the tumor site.^39,151–156^ Consequently, only a limited fraction of tumor cells undergo ICD in situ, often without sufficient activation of innate immune pathways. Moreover, only a subset of chemotherapeutics triggers the stress responses necessary for ICD. For instance, doxorubicin and oxaliplatin induce eIF2α phosphorylation and oxidative stress, whereas paclitaxel or cisplatin frequently fail to elicit adequate ER stress or ROS, resulting in weak DAMP emission despite inducing tumor cell death.^157^ These limitations underscore that the distinction between cytotoxicity and immunogenicity, cell death alone is not sufficient unless accompanied by appropriate immunostimulatory signals.

Tumor-intrinsic resistance mechanisms also play a central role. Many cancer cells adapt to stressful conditions by upregulating stress-buffering proteins such as glucose-regulated protein 78 (GRP78),^158^ which dampens ER stress signaling, or activating nuclear factor erythroid 2–related factor 2 (Nrf2)-mediated antioxidant pathways to suppress ROS accumulation,^159^ thereby blunting DAMP release even in response to ICD-inducing agents. Radiotherapy, PDT, and sonodynamic therapy (SDT) primarily induce cell death through the generation of high levels of intracellular ROS.^160^ Elevated concentrations of ROS can disrupt subcellular organelles, including mitochondria, ER, and lysosomes, leading to cellular dysfunction and eventual cell death,^161,162^ leading to the release of DAMPs, TAAs, proinflammatory cytokines, and chemokines. These events can stimulate tumor-specific T-cell responses and ultimately induce ICD.^163^ However, these treatments frequently fail to fully activate PRRs such as TLRs or the STING pathway in DCs, resulting in suboptimal APC maturation and impaired T-cell priming.^164^ The absence of coordinated innate activation during antigen release remains a key bottleneck.

The immunological outcome of ICD is also profoundly influenced by the cellular context in which DAMPs are sensed. While DAMP recognition by PRRs on DCs promotes antigen uptake and T-cell priming, the same PRRs, when expressed on tumor cells, can elicit opposing effects.^165^ Activation of TLR7, TLR8, or TLR9 in cancer cells has been shown to enhance chemoresistance, survival, and metastatic potential via NF-κB activation and upregulation of antiapoptotic proteins such as Bcl-2.^166^ Tumor-intrinsic or acquired resistance mechanisms further impair ICD induction. Cancer cells often evade immunogenic death by overexpressing antiapoptotic proteins (e.g., Bcl-2 and myeloid cell leukemia-1 (Mcl-1)), disabling apoptotic signaling pathways, or upregulating drug efflux transporters such as P-glycoprotein (MDR1).^167–170^ Additionally, host genetic polymorphisms can influence ICD efficacy. Loss-of-function variants in DAMP sensors such as TLR4 (Asp299Gly), purinergic receptor P2X, and ligand-gated ion channel 7 (P2RX7) (Glu496Ala) are associated with poor responses to anthracyclines and platinum-based chemotherapy due to impaired innate immune activation following DAMP release.^57,171,172^

Finally, the metabolic composition of the TME exerts significant immunosuppressive pressure. Although ATP acts as a potent “find-me” signal during ICD, it is rapidly hydrolyzed into adenosine by the ectoenzymes CD39 and CD73.^173^ Adenosine, in turn, binds to A2A receptors on immune cells to suppress cytokine production, inhibit cytotoxicity, and block T-cell proliferation, thereby limiting the efficacy of ICD inducers such as doxorubicin and oxaliplatin.

The key limitation of conventional ICD strategies lies in their inability to consistently pair antigen release with a sufficiently immunogenic context. Overcoming this challenge will require integrative approaches that address delivery constraints, circumvent tumor cell-intrinsic resistance, modulate immunosuppressive metabolism, and account for host genetic variability. Only by ensuring that tumor cell death is accompanied by effective innate activation and T-cell priming can in situ vaccination strategies reach their full therapeutic potential.

### Immunosuppressive TME

**Fig. 6:**
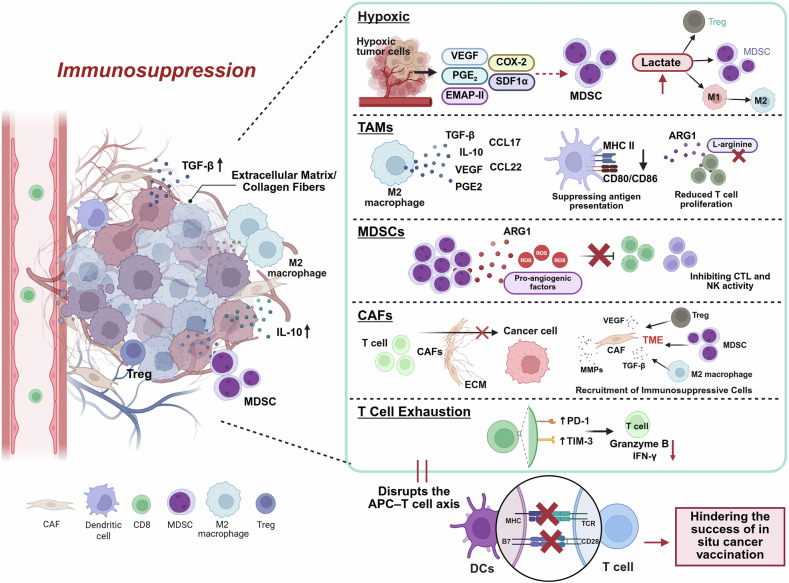
The immunosuppressive tumor microenvironment limits the efficacy of in situ cancer vaccination. The immunosuppressive tumor microenvironment (TME) is characterized by hypoxia, immunosuppressive cell populations (TAMs, MDSCs, Tregs), and extracellular matrix barriers formed by CAFs. Hypoxia-induced factors (VEGF, COX-2, PGE₂, SDF1α) recruit MDSCs and polarize macrophages toward an M2 phenotype. TAMs and MDSCs release IL-10, TGF-β, and ARG1, suppressing dendritic-cell maturation and T-cell proliferation, while CAFs create a physical barrier that impedes immune infiltration. Together, these processes disrupt the APC–T-cell axis, promote T-cell exhaustion, and hinder the success of in situ cancer vaccination. The figure was generated with BioRender (https://biorender.com)

As with other tumor immunotherapies, the efficacy of in situ cancer vaccination is constrained by the immunosuppressive tumor microenvironment (TME). The development of a highly immunosuppressive TME represents a universal barrier across cancer immunotherapies, limiting durable antitumor immune responses. Solid tumors create an immunosuppressive milieu characterized by immune checkpoint activation,^174^ suppressive immune cells (TAMs, MDSCs, Tregs), and inhibitory cytokines (e.g., IL-10, TGF-β). These mechanisms collectively lead to antigen-specific T-cell exhaustion or inactivation, enabling tumor immune evasion. Within the context of in situ cancer vaccines, this challenge is particularly critical. The therapeutic principle of in situ cancer vaccination relies on efficient antigen capture by local APCs, their maturation, and subsequent migration to lymph nodes for T-cell priming. However, the immunosuppressive TME disrupts this APC–T-cell axis at multiple levels, including DC activation, trafficking, and antigen presentation, thereby undermining the generation of robust cytotoxic T-cell responses (Fig. 6).

Hypoxia, induced by tumor growth and abnormal vasculature, reshapes the TME into a tolerogenic niche. Hypoxic tumor cells release vascular endothelial growth factor (VEGF), cyclooxygenase-2 (COX-2), prostaglandin E₂ (PGE₂), stromal cell-derived factor 1 alpha (SDF1α), and endothelial monocyte-activating polypeptide II (EMAP-II), recruiting and maintaining immunosuppressive myeloid cells. SDF1α binding to CXCR4 on MDSCs enhances their retention and function, while the Warburg effect leads to lactate buildup, promoting M2 polarization of TAMs, Treg expansion, and further MDSC differentiation. These cells release IL-10 and TGF-β, which suppress DC maturation and function, ultimately impairing CD8⁺ T-cell priming. Beyond lactate accumulation, hypoxia itself acts as a potent immune suppressant by impairing effector T-cell metabolism and APC function. Under hypoxic stress, the stabilization of hypoxia-inducible factor 1α (HIF-1α) limits oxidative phosphorylation and promotes glycolytic exhaustion in CD8⁺ T cells,^175,176^ reducing their proliferation, cytokine production (e.g., IFN-γ, granzyme B), and cytotoxicity. Simultaneously, hypoxia impairs DC differentiation and antigen presentation by downregulating MHC class II and costimulatory molecules (CD80/CD86),^177^ as well as suppressing IL-12 secretion, thereby weakening T-cell activation. Pharmacological strategies such as proton pump inhibitors^178^ have been shown to alleviate hypoxia-driven immunosuppression, restore antitumor T-cell activity, and improve therapeutic efficacy.^178^

TAMs, especially M2-skewed subsets, secrete immunosuppressive mediators (IL-10, TGF-β, VEGF, PGE2, C–C motif chemokine ligand 17 (CCL17), and C–C motif chemokine ligand 22 (CCL22)),^179–181^ suppressing antigen presentation by downregulating MHC-II and CD80/CD86. They also express arginase-1 (ARG1), depleting L-arginine, which is essential for TCR signaling, leading to reduced T-cell proliferation.^182–184^ Tregs, recruited by TAM-derived chemokines, inhibit DCs and CD8⁺ T cells through IL-10-, TGF-β-, CTLA-4-mediated blockade, IL2 consumption, and granzyme/perforin-induced apoptosis. MDSCs further suppress CTL and NK cells via ARG1, ROS, and proangiogenic factors.^185–187^ Collectively, these mechanisms reduce T-cell proliferation and cytokine secretion and increase the expression of inhibitory receptors such as PD-1 and T-cell immunoglobulin and mucin-domain containing-3 (TIM-3).^188,189^ Even when in situ vaccination activates tumor-specific T cells, their antitumor activity is quickly suppressed upon re-entry into the TME, leading to functional exhaustion and immune failure.

T-cell responses, central to adaptive immunity, are also compromised in the TME. These immunosuppressive mechanisms are compounded by physical barriers, particularly those orchestrated by cancer-associated fibroblasts (CAFs). CAFs secrete VEGF, matrix metalloproteinases (MMPs), and TGF-β to promote angiogenesis, ECM remodeling, and recruitment of immunosuppressive cells.

In this context, the immunosuppressive TME acts as a major bottleneck to the efficacy of in situ cancer vaccination. Although in situ cancer vaccines induce the release of tumor-associated antigens and danger signals through local ICD induction, these signals are often neutralized by the suppressive cytokine milieu and inhibitory cell populations within the TME. The impaired DC activation and T-cell priming caused by hypoxia, TAMs, MDSCs, and Tregs collectively diminish the potency of the vaccine-induced immune response. Consequently, in situ cancer vaccines require adjuvant systems capable of reprogramming the TME from an immunosuppressive to an immunostimulatory state. Potent immunoadjuvants used in in situ cancer vaccines have been shown to downregulate MDSCs, M2-type TAMs, Tregs, and immature DCs while promoting the infiltration and activation of CD8⁺ T cells, mature DCs, and M1 macrophages. This bidirectional remodeling converts the TME from a cold and tolerogenic niche into a hot and inflamed environment, restoring the APC–T-cell axis and enabling robust antitumour immunity. Through this mechanism, in situ cancer vaccines not only elicit local tumor control but also drive systemic immune activation, contributing to durable antitumor protection and potential abscopal effects.

In conclusion, the immunosuppressive TME interferes with in situ cancer vaccination at multiple levels: disrupting DC activation and maturation, impairing antigen transport to lymph nodes, inducing T-cell exhaustion, and creating physical exclusion via CAFs. These interdependent barriers ultimately undermine the APC–T-cell axis essential for generating durable and effective antitumour immunity.

### Delivery and retention barriers of therapeutic agents

Although intratumoral administration can concentrate agents at the disease site, the physical transport properties of solid tumors frequently impede uniform distribution and durable retention of in situ cancer vaccination components. Elevated interstitial fluid pressure (IFP) and abnormal vasculature reduce convective transport,^190,191^ favor outward fluid flux, and create steep gradients that keep drugs near injection tracts or tumor peripheries. High IFP is closely associated with poor macromolecule penetration and patchy intratumoral coverage after local injection.^192^

The tumor extracellular matrix (ECM), primarily composed of dense collagen fibers, hyaluronan, fibronectin, periostin, and other macromolecules, forms a physical and biochemical barrier that significantly limits the penetration and distribution of therapeutic agents, including nanoparticles, cytokines, and PRR agonists.^193^ The dense and cross-linked collagen networks, together with elevated solid stress, create a rigid and heterogeneous structure that restricts convective transport and molecular diffusion within the tumor microenvironment.^193^ Recent analyses have shown that ECM remodeling is not a static process but a dynamic niche that evolves with tumor progression, simultaneously supporting cancer cell proliferation, invasion, and immune evasion while also hindering drug accessibility.^194^ Structural reorganization of the ECM, such as collagen fiber thickening, linearization, and alignment, further exacerbates these transport barriers by increasing tissue stiffness and reducing pore sizes available for drug diffusion.^194^

In addition, tumor vascular abnormalities significantly influence delivery outcomes.^195^ The dense structure of the tumor mass impairs therapeutic agent penetration, while the aberrant architecture and variable pore sizes of the tumor endothelium across primary and metastatic sites or even within the same tumor type pose substantial challenges for passive transport.^196^ To achieve therapeutic concentrations at the tumor site, drug doses often need to be escalated toward maximum tolerated levels to generate sufficient blood–tumor concentration gradients.^197^ This strategy, however, not only fails to guarantee uniform drug absorption but also exacerbates systemic toxicity.

Finally, practical issues in intratumoral administration compound these biophysical barriers. Needle placement, injection rate, and pressure can influence reflux, leakage, and depot geometry.^198^ Even under image guidance, postinjection imaging frequently reveals variable intralesional distribution and unintended extratumoral spillage.^198^ Collectively, these factors result in inconsistent exposure of dendritic cells and tumor cells to ICD inducers or adjuvants, limiting the potency and reproducibility of in situ cancer vaccination across patients and tumor lesions.

## Enhancing in situ vaccination; a combinational approach

Nanomedicine has emerged as a versatile and powerful platform to overcome the intrinsic limitations of conventional in situ cancer vaccines. By integrating principles of targeted delivery, spatiotemporal control, and multifunctional payload design, nanoplatforms significantly enhance antigen release, immune activation, and retention within the TME. Targeted delivery enables precise accumulation of immunotherapeutic agents at tumor sites through passive enhanced permeability and retention (EPR) effects or active targeting ligands such as antibodies, peptides, or polysaccharides, thereby minimizing systemic exposure, reducing off-target toxicity, and maximizing antigen and adjuvant availability to tumor resident APCs. Moreover, targeted nanocarriers can be engineered to interact with specific immune or stromal cell subsets, such as dendritic cells, macrophages, or tumor-associated fibroblasts, reprogramming the local immune landscape toward an immunostimulatory phenotype. Spatiotemporal control provided by nanomedicine ensures synchronized and sustained immune activation through stimuli-responsive materials that release immunomodulators in response to environmental triggers such as pH, reactive oxygen species, enzymes, or external cues such as light or heat. This precise regulation prolongs antigen presentation and DAMP/PAMP signaling while maintaining immune engagement and avoiding premature clearance or excessive inflammation. Multifunctional payload integration allows nanocarriers to codeliver diverse therapeutic agents, including ICD inducers, immunoadjuvants, checkpoint inhibitors, and cytokines, within a single delivery system, ensuring synergistic activation of innate and adaptive pathways and facilitating coordinated crosstalk between dying tumor cells and immune effector populations. Collectively, these mechanistic advantages endow nanomedicine platforms with superior capability to amplify the antigenicity and adjuvanticity of dying tumor cells, achieve precise immune modulation within the TME, and synergize effectively with immune checkpoint blockade (ICB), ultimately leading to more robust and durable antitumor immunity.

### Synergy with immune checkpoint blockade

In situ cancer vaccination primes broad, patient-specific T-cell responses by releasing diverse tumor antigens and activating antigen-presenting cells, but the efficacy is often limited by immune checkpoints such as PD-1/PD-L1 and CTLA-4 that suppress CTL activity within the TME. Combining in situ cancer vaccination with ICB can overcome this barrier, sustaining effector T-cell function and transforming “cold” tumors into “hot” tumors with dense immune infiltration and proinflammatory signaling.^76,199,200^

The combination of an in situ cancer vaccine with immune checkpoint blockade addresses tumor immune escape by targeting both the diversity of the antitumor immune response and the functional exhaustion of T cells. In situ cancer vaccines initiate a broad polyclonal T-cell response by exposing the immune system to a wide array of tumor antigens released from local tumor cell death, thereby reducing the likelihood that antigen-loss variants can evade immune detection since multiple tumor neoantigens are targeted simultaneously. However, tumor progression often involves clonal evolution, leading to antigenic heterogeneity that can outpace immune surveillance. Immune checkpoint blockade complements in situ cancer vaccines by reversing T-cell exhaustion caused by chronic antigen stimulation and immunosuppressive signals within the tumor microenvironment. By restoring effector function and proliferative capacity in tumor-infiltrating lymphocytes, checkpoint inhibitors maintain and amplify the polyclonal T-cell response primed by the in situ cancer vaccine. Together, these therapies help sustain immune pressure across a diverse set of tumor clones, limiting the emergence and expansion of resistant antigen-loss variants and preventing immune escape driven by clonal evolution. Thus, this combinational approach enhances both the breadth and durability of antitumor immunity, offering a robust strategy to overcome tumor immune evasion.

Nanomedicine platforms have advanced this synergy by enabling colocalized and sustained intratumoral delivery of adjuvants, tumor antigens, and checkpoint inhibitors, thereby enhancing local immune activation while reducing systemic toxicity.^201,202^

Duan et al. developed a self-assembled nanoscale coordination polymer (NCP) core–shell nanoparticle (OxPt/DHA) for the codelivery of oxaliplatin (OxPt) and dihydroartemisinin (DHA), enabling selective accumulation in colorectal cancer (CRC) tumors with controlled drug release and minimized systemic toxicity.^203^ The combination significantly amplified reactive oxygen species (ROS) generation, causing mitochondrial dysfunction, cytochrome c release, and apoptosis/necrosis. Compared to monotherapy, OxPt/DHA increased early apoptotic cells by ~2.2-fold and late apoptotic/necrotic cells by ~2.3-fold, confirming synergistic cytotoxicity. In CT26 tumor-bearing mice, low-dose α-PD-L1 monotherapy showed negligible efficacy, while OxPt/DHA plus α-PD-L1 achieved complete tumor regression in all treated mice within 40–50 days and prevented recurrence for at least 120 days, indicating durable tumor-specific immune memory. In the more immunosuppressive MC38 model, OxPt/DHA alone delayed tumor growth, and combination therapy with α-PD-L1 extended tumor control, with dose escalation achieving complete responses in 3 of 5 mice and long-term survival. Tumor rechallenge experiments demonstrated that mice previously cured with combination therapy were fully protected against CT26 cells but not 4T1 cells, confirming tumor antigen-specific immune memory. OxPt/DHA plus α-PD-L1 markedly increased intratumoral CD8⁺ T-cell infiltration by ~4-fold compared to ~1-fold with OxPt/DHA alone, while CD45⁺ leukocytes and CD4⁺ T cells remained unchanged. The combination also enhanced dendritic-cell (CD11c⁺) and macrophage (F4/80⁺) recruitment, with a shift toward the proinflammatory M1 phenotype, facilitating antigen presentation and T-cell activation.

Kuai et al. developed a nanodisc-based chemoimmunotherapy platform leveraging synthetic high-density lipoprotein (sHDL)–mimicking nanodiscs to deliver doxorubicin (DOX), a chemotherapeutic known to induce ICD. These ultrasmall (~10 nm) nanodiscs encapsulate DOX and stably release it in acidic endosomal environments, markedly improving pharmacokinetics and tumor accumulation compared to free DOX.^204^ Treated cancer cells exhibited hallmark ICD features, including ~3.5-fold increased CRT exposure and ~4-fold higher HMGB1 release compared to free DOX. This potent immunogenic priming translated into robust CD8⁺ T-cell activation, with the frequency of tumor antigen-specific CD8⁺ T cells increasing by ~6-fold in tumor-draining lymph nodes. Combination chemoimmunotherapy using sHDL nanodiscs loaded with doxorubicin (sHDL-DOX) in combination with anti–PD-1 therapy-induced complete regression of established CT26 and MC38 colon carcinoma tumors in 80–88% of treated mice and generated durable immune memory, far surpassing either monotherapy. Moreover, mice that achieved complete responses developed long-term immune memory, resisting tumor rechallenge for over 60 days, confirming that this nanodisc-based priming strategy elicits broad, durable antitumor immunity.

Moon et al. engineered anti–PD-L1 peptide–conjugated prodrug nanoparticles (PD-NPs) that simultaneously deliver doxorubicin (DOX) to induce ICD and block PD-L1–mediated immune suppression within tumors. These nanoparticles were self-assembled from a multifunctional peptide (anti–PD-L1 sequence CVRARTR linked via a cathepsin B–cleavable linker to a DOX prodrug, FRRG) and DOX molecules.^205^ The resulting PD-NPs accumulated in tumor tissue through the EPR effect and were internalized via PD-L1–mediated endocytosis. Upon exposure to intratumoral cathepsin B, the conjugates released DOX, triggering strong ICD characterized by DAMP release, calreticulin exposure, and enhanced dendritic-cell activation. Concurrently, PD-L1 blockade occurred via nanoparticle-mediated lysosomal degradation, effectively reinvigorating tumor-infiltrating T lymphocytes. In 4T1 murine breast tumor-bearing BALB/c mice, median survival improved to >30 days with PD-NPs, compared to ~20 days for free DOX, ~16 days for free DOX + anti–PD-L1, and ~18 days for saline, highlighting the platform’s superior therapeutic benefit. Importantly, PD-NPs minimized systemic toxicity, maintained stable body weights, and showed no significant histopathological abnormalities in major organs.

Collectively, these findings underscore that nanomedicine-enabled in situ cancer vaccination combined with ICB represents a rational and potent strategy to elicit durable systemic antitumor immunity and overcome resistance to monotherapies.

### Effective ICD induction

Evidence suggests that the efficacy of ICD inducers is largely determined by the immune phenotype of the tumor. In immunologically “hot” tumors characterized by preexisting immune cell infiltration, conventional chemotherapeutic agents such as oxaliplatin and doxorubicin effectively potentiate antitumor immune responses by triggering ICD, thereby promoting the release of DAMPs that facilitate dendritic-cell activation and subsequent T-cell priming.^206,207^ For instance, anthracyclines, including doxorubicin, have been shown to enhance CD8⁺ T-cell and NK cell activity in neuroblastoma spheroid models, increasing IFN-γ and granzyme B production.^208^ In cold tumors, characterized by minimal immune infiltration and strong immunosuppressive barriers, more potent strategies are required to reprogram the TME and initiate immune activation. Radiotherapy combined with STING agonists, especially when delivered via nanomedicine for improved stability and targeting, has demonstrated enhanced immunogenicity and TME reprogramming.^209–211^ Similarly, ferroptosis-inducing nanoplatforms, such as Mn(III)-based nanoenzymes, can trigger ICD through DAMP release, activate the cGAS–STING pathway, and recruit CD8⁺ T cells, creating a positive immune feedback loop.^212^ These insights emphasize that tailoring ICD strategies to the tumor’s immune phenotype is essential for effective immune activation and durable responses. Building on this understanding, recent advances in nanomedicine have leveraged these principles to refine ICD-based in situ cancer vaccines, enhancing antigenicity and adjuvanticity while overcoming key barriers in conventional approaches.

With the advancement of cancer immunotherapy, ICD-based in situ cancer vaccines have emerged as a promising therapeutic approach. Nanomedicine addresses key limitations of ICD-based in situ cancer vaccines by enhancing tumor antigenicity and adjuvanticity through targeted nanotechnological platforms. This strategy amplifies the antigenicity and adjuvanticity of dying tumor cells, facilitating efficient DC maturation and robust T-cell priming. The following examples highlight recent advances in nanotechnology-based ICD induction strategies that address the limitations of conventional in situ cancer vaccines and demonstrate promising antitumor efficacy across various preclinical and clinical models.

Wang et al. developed mannosylated lactoferrin nanoparticles (Man-LF NPs) for the codelivery of shikonin (SHK), a potent ICD inducer, and JQ1, a small-molecule bromodomain and extraterminal (BET) inhibitor that targets BRD4 and is known to downregulate PD-L1.^213^ The mannose decoration enabled dual targeting of tumor cells (via low-density lipoprotein receptor–related protein 1 (LRP-1)) and tumor-associated macrophages (via mannose receptors), improving tumor accumulation and immune modulation. This platform induced hallmark features of ICD, including CRT exposure, HMGB1 release, and ATP secretion, and effectively reprogrammed immunosuppressive TAM2 cells toward a proinflammatory TAM1 phenotype while remodeling tumor glucose metabolism and downregulating PD-L1 expression. ICD induction translated into potent immune activation: coculture assays revealed that Man-LF NPs induced approximately 83% dendritic-cell maturation (CD80⁺CD86⁺), significantly outperforming nontargeted NPs and free-drug formulations. In CT26 tumor-bearing mice, Man-LF NPs achieved ~84% tumor growth inhibition and extended median survival to 44 days, markedly superior to LF NPs and free SHK/JQ1. Treatment further enhanced DC maturation, boosted CD8⁺ T-cell infiltration, reduced Treg accumulation, and elevated intratumoral IFN-γ and TNF-α levels, demonstrating profound tumor immune microenvironment (TIME) remodeling and durable antitumor immunity.

To activate pyroptosis, Li et al. successfully developed lipid nanoparticles (LNPs) encapsulating a single-agent mRNA encoding the GSDMB N-terminal domain (GSDMB^NT^ mRNA@LNPs), which trigger inflammatory GSDM-mediated pyroptosis to turn cold tumors hot.^62^ GSDMB^NT^ mRNA@LNPs were injected intratumorally into BALB/c mice to treat an orthotopic 4T1 breast tumor model, and both TNF-α and IFN-γ levels in serum and tumor tissue remained elevated for over 72 h. Moreover, treatments with GSDMB^NT^ mRNA@LNPs significantly inhibited tumor growth and extended survival by 8 days relative to controls.

Liang et al. developed an MUC1 aptamer-targeted nanocomplex (MUC1@Chi-Ag@CPB@SHK, abbreviated as MUC1@ACS) for codelivering shikonin (SHK) and chitosan silver nanoparticles (Chi-Ag NPs).^214^ The acid-responsive release of SHK and Chi-Ag NPs from MUC1@ACS NPs cooperatively induced necroptosis in tumor cells by upregulating the expression of receptor-interacting serine/threonine-protein kinase 3 (RIPK3), phosphorylated RIPK3 (p-RIPK3), and tetrameric mixed lineage kinase domain-like protein (MLKL), thereby effectively triggering ICD. They demonstrated that MUC1@ACS effectively induced RIPK3-dependent necroptosis in triple-negative breast cancer (TNBC) cells.

Multipathway ICD strategies address the major limitations of conventional single-mechanism inducers by simultaneously activating complementary forms of regulated cell death. This enhances the diversity of DAMP release, overcomes tumor-intrinsic resistance, promotes broader antigen presentation and epitope spreading, and amplifies synergy with immune adjuvants or checkpoint blockade, ultimately leading to more robust and durable antitumor immunity.

Hou et al. developed hydrazided hyaluronic acid (HHA)–modified Zn–CuO₂ (HZCO) nanoparticles that induce PANoptosis by disrupting intracellular ion homeostasis.^215^ Upon internalization by tumor cells, HZCO releases Cu²⁺ and Zn²⁺ in response to the tumor microenvironment, triggering mitochondrial dysfunction, ER stress, and the accumulation of ROS and cytosolic dsDNA. These stress signals activate the AIM2 inflammasome and PANoptosome complex, leading to the concurrent activation of pyroptosis (caspase-1/GSDMD), apoptosis (caspase-8), and necroptosis (RIPK3/MLKL). In vivo, intratumoral administration of HZCO markedly suppressed tumor growth in 4T1 breast cancer, CT26 colorectal, and B16F10 melanoma models. These findings demonstrate the broad-spectrum antitumor efficacy of HZCO through the coordinated induction of immunogenic PANoptosis.

Pexa-Vec (JX-594), a genetically engineered oncolytic vaccinia virus expressing GM-CSF, exemplifies a virus-based nanomedicine designed to induce ICD and potentiate antitumor immunity.^216^ In phase I/II trials for metastatic colorectal cancer, Pexa-Vec combined with checkpoint inhibitors showed a favorable safety profile and immune activation, including increased Ki67⁺ CD8⁺ T-cell proliferation and tumor microenvironment modulation. Pexa-Vec mediates selective oncolysis, releasing DAMPs and TAAs, thereby functioning as an in situ cancer vaccine. However, clinical efficacy remains limited due to neutralizing antibodies and restricted T-cell infiltration, highlighting the need for optimized combination strategies.

NK012 is a polymeric micelle-based nanotherapeutic encapsulating 7-ethyl-10-hydroxycamptothecin (SN-38), the active metabolite of irinotecan, engineered to improve pharmacokinetics and tumor-selective delivery via the EPR effect.^217,218^ Released SN-38 induces DNA damage and apoptosis, triggering ICD-associated immune activation. In a phase I/II clinical study, NK012 exhibited a manageable toxicity profile and achieved a disease control rate of 56.6% and a median overall survival of 15.0 months in metastatic colorectal cancer, with reduced irinotecan-related severe diarrhea. These results support NK012 as a promising ICD-inducing nanomedicine for combination immunotherapy.

Lurbinectedin (PM01183) is a second-line therapy developed by PharmaMar that received FDA approval for the treatment of adult patients with metastatic small-cell lung cancer (SCLC) who have relapsed after first-line platinum-based chemotherapy.^219,220^ As a DNA-binding transcriptional inhibitor, it potently induces ICD in tumor cells. The drug activates the cyclic guanosine monophosphate-adenosine monophosphate synthase-stimulator of interferon genes (cGAS–STING) pathway,^219,220^ leading to interferon signaling, upregulation of proinflammatory chemokines (CCL5 and CXCL10), and increased expression of MHC-I and ICD-associated DAMPs.^221^ It enhances CD8⁺ T-cell infiltration, promotes M1 macrophage polarization, and synergizes with PD-1/PD-L1 blockade to elicit durable antitumor immunity.^219,222^ These findings establish lurbinectedin as a clinically validated ICD inducer that enhances anticancer immunity via activation of the STING–IFN axis.^219,220^

### Remodeling the immunosuppressive TME

Tumor hypoxia and elevated reactive oxygen species (ROS) are two hallmark features of the TME that jointly contribute to immunosuppression and therapeutic resistance. Hypoxia hampers CTL activity and undermines ROS-dependent therapies such as photodynamic therapy (PDT), which also restricts the generation of DAMPs during ICD. To address this, oxygen-generating or oxygen-carrying nanoplatforms have been developed to locally relieve hypoxia and potentiate ICD-driven immune activation.^223–225^

In a study by Yang et al.,^226^ hollow silica nanoparticles encapsulating catalase (CAT) and chlorin e6 (Ce6) were developed to catalyze endogenous H_2_O_2_ into oxygen, thereby relieving hypoxia and enhancing PDT efficacy. The nanoparticles were further functionalized with a mitochondrial-targeting moiety (CTPP) and coated with a pH-responsive, charge-convertible polymer for improved tumor penetration and cellular uptake. In vivo, this system exhibited significant tumor suppression in 4T1 tumor-bearing mice following laser irradiation, outperforming control groups treated with free Ce6 or nontargeted nanoparticles.

Similarly, He et al. engineered a hypoxia/photoresponsive nanoplatform composed of polyethyleneimine-nitroimidazole (PEI-NI) micelles coassembled with Ce6-conjugated hyaluronic acid (HC).^227^ The nitroimidazole moiety acted as a hypoxia-sensitive trigger that underwent bioreduction in low-oxygen environments to facilitate doxorubicin release, while Ce6 generated reactive oxygen species (ROS) under laser irradiation. In vivo, this dual-responsive system exhibited significant antitumor efficacy in Lewis lung carcinoma (LLC) xenograft tumor-bearing mice, resulting in substantial tumor volume reduction and increased apoptosis compared to free DOX or single-component controls. This dual activation synergistically promoted dendritic-cell activation and CD8⁺ T-cell priming, translating into robust tumor growth suppression in Lewis lung carcinoma models.

Tumor-associated macrophages (TAMs) are another critical driver of immunosuppression, often polarized toward the M2 phenotype that fosters angiogenesis, tumor progression, and immune evasion. Reprogramming TAMs to the proinflammatory M1 phenotype enhances antigen presentation, cytokine production (TNF-α, IL-12), and recruitment of effector CD8⁺ T cells, thereby reinforcing ICD-driven immune activation. Nanostructure-based systems provide an effective means to deliver immunomodulatory agents or stimuli directly to TAMs, enabling targeted immune re-education.

In a study by Cao et al.,^228^ naturally occurring ginseng-derived nanoparticles (GDNPs), isolated from Panax ginseng roots, were shown to reprogram M2-like TAMs to the M1 phenotype via the Toll-like receptor 4 (TLR4)/MyD88 signaling pathway. These EV-like nanoparticles, enriched in proteins, lipids, and nucleic acids, activated TLR4 signaling in macrophages, promoting NF-Κb-mediated transcription of proinflammatory mediators. GDNPs significantly suppressed IL-4/IL-13-induced M2-like polarization, as evidenced by downregulation of M2 markers such as CD206 and reduced expression of M2-related genes, while enhancing secretion of M1-associated cytokines, including TNF-α, IL-12, and IL-6, suggesting a Th1-biased immune environment favorable for CD8⁺ T-cell activation. GDNP monotherapy significantly suppressed tumor growth in B16F10 melanoma-bearing mice through sustained M1-like macrophage polarization and enhanced antitumor immune activity.

In another strategy to overcome both drug resistance and TAM-mediated immunosuppression in breast cancer, Xie et al. developed a furin-responsive gold nanoparticle system (AuNPs-D&H-R&C).^229^ This platform exploits overexpression of the protease furin in the tumor microenvironment to trigger in situ nanoparticle aggregation, enhancing tumor retention via the enhanced permeability and retention (EPR) effect. The nanoparticles, coloaded with doxorubicin and hydroxychloroquine (HCQ), released their cargo within acidic lysosomes, where doxorubicin exerted cytotoxic effects, and HCQ blocked protective autophagy and reprogrammed M2-like TAMs to the M1 phenotype by activating NF-κB and increasing TNF-α and IL-6 secretion. This dual-function approach synergistically suppressed tumor growth in MCF-7/ADR chemoresistant breast cancer xenograft models by overcoming doxorubicin resistance and reversing the immunosuppressive tumor microenvironment.

Wei et al.^230^ combined doxorubicin-elicited ICD with TAM-targeted immunomodulation using a hybrid bacterial-nanoparticle platform. PLGA nanoparticles carrying the TLR7/8 agonist R848 (PR848) were anchored onto engineered *E. coli* MG1655 via glycol chitosan (Ec-PR848), while doxorubicin was delivered separately using PLGA nanoparticles (PDOX). R848, a well-characterized TLR7/8 agonist, reprograms M2-like TAMs into proinflammatory M1-like macrophages by activating the MyD88–NF-κB signaling pathway, inducing the secretion of TNF-α and IL-6. *E. coli* components such as lipopolysaccharide (LPS) and flagellin further contribute to macrophage repolarization by engaging TLR4 and TLR5. In a 4T1 breast cancer model, Ec-PR848 + PDOX significantly suppressed tumor growth by enhancing TAM repolarization and ICD induction and promoting dendritic-cell activation, CD8⁺ T-cell infiltration, and antitumor immunity.

### Boosting innate-adaptive immune crosstalk

Efficient crosstalk between innate sensing and adaptive immunity is a critical determinant of successful in situ vaccination. While tumor-associated antigens can be released via ICD, the absence of robust innate immune activation often leads to suboptimal DC maturation and insufficient T-cell priming. To address this, synthetic agonists targeting PRRs, such as TLRs,^231^ NLRs,^100^ and stimulator of interferon genes (STING),^232^ have been developed to activate innate immune pathways and enhance antigen processing and presentation. However, the dense extracellular matrix, high interstitial pressure, and abnormal vasculature of tumors hinder the efficient delivery of PRR agonists. Moreover, free immunostimulatory adjuvants often diffuse into healthy tissues, leading to systemic toxicity. To circumvent these challenges, nanocarrier-based delivery strategies have been developed to improve localization, minimize toxicity, and enhance immunomodulatory efficacy.

To improve postablation immune activation, van den Broek et al. investigated the use of CpG oligodeoxynucleotides (ODN 1668),^233^ a TLR9 agonist, as an immunoadjuvant following cryoablation in a murine B16 melanoma model. Intratumoral CpG injection after cryoablation significantly enhanced tumor control, reducing recurrence from 30% to 0%, and 50% of mice rejected both the primary tumor and a subsequent B16F10 challenge, indicating durable immune memory. In a lung metastasis model, cryoablation plus CpG also caused regression of metastatic lesions. CpG-ODNs activate plasmacytoid dendritic cells and B cells via the TLR9–MyD88–NF-κB pathway, inducing CD80/CD86 expression and cytokine secretion (e.g., type I interferons, IL-12), which enhance T-cell priming and systemic antitumor immunity.

To further improve delivery and spatial control, Li et al. engineered black phosphorus nanovesicles (BPNVs) encapsulating CpG oligodeoxynucleotides for near-infrared (NIR)-triggered photodynamic immunotherapy.^234^ Upon NIR laser irradiation, the BPNVs disassembled into ultrasmall black phosphorus quantum dots (BPQDs), enhancing tumor penetration and enabling controlled release of CpG. This treatment-induced calreticulin (CRT) exposure on tumor cells, upregulated maturation markers on dendritic cells (CD80⁺, MHC-II⁺), and increased infiltration of CD8⁺ T cells into tumors. In vivo, BPNVs–CpG therapy in a bilateral 4T1 breast cancer model in BALB/c mice significantly suppressed both primary and untreated distant tumors, reduced lung metastases, and elevated systemic cytokine levels (TNF-α, IL-6, IL-12), demonstrating a potent abscopal effect (Table 3).

**Table 3:** Representative PRR agonists and their nanocarrier-based delivery strategies for immune activation in in situ cancer vaccination

PRR type	Representative agonist (PAMP)	Nanocarrier system	Target cell/effect	Immune activation outcome
TLR9	CpG oligodeoxynucleotides	PLGA nanoparticles, dendrimers, gold NPs	DCs	↑ DC maturation, ↑ Th1 cytokines (e.g., IFN-α), ↑ CTL activation
TLR4	Monophosphoryl lipid A (MPLA)	Liposomes, lipid nanoparticles	DCs, macrophages	↑ NF-κB signaling, ↑ cross-presentation, ↓ systemic toxicity
CLR (e.g., DC-SIGN, MMR)	Mannan	Mannose-modified liposomes or polymeric NPs	TAMs, DCs	TAM reprogramming (M2 → M1), ↑ antigen uptake, ↑ IL-12
STING (cytosolic DNA sensor)	Cyclic dinucleotides (CDNs, e.g., cGAMP)	Lipid-based NPs, pH-sensitive vesicles	DCs, macrophages	T cells
TLR3	Poly(I:C) (dsRNA analog)	Cationic liposomes, polymeric micelles	DCs (especially cDC1)	↑ IFN-β, ↑ cross-priming of CD8 + T cells
TLR7/8	Imiquimod, R848 (Resiquimod)	Hydrogel depot, nanoemulsions	DCs, monocytes	↑ TNF-α, IL-6, enhanced local inflammation and CTL priming

Parallel strategies have sought to codeliver PRR agonists and tumor antigens using multifunctional nanoparticles. Baljon et al. developed pH-responsive polymeric nanoparticles encapsulating MPLA (a TLR4 agonist), cGAMP (a STING agonist), and tumor-specific peptides.^235^ These particles promoted endosomal escape and STING-TBK1-IRF3 pathway activation, resulting in increased secretion of IFN-β and IL-12 by bone marrow–derived DCs. The system enhanced cross-presentation by CD103⁺ DCs in draining lymph nodes and expanded tumor-specific CD8⁺ T cells by over threefold, leading to complete tumor regression in >60% of mice. For example, the nanoSTING-vax platform developed by Shae et al. uses pH-responsive polymersomes to codeliver peptide neoantigens and a STING agonist (cGAMP). This coordinated delivery amplifies dendritic-cell activation and cross-presentation, leading to robust CD8⁺ T-cell priming, enhanced antitumor immunity, and the generation of durable immunological memory.^236^

To enhance the magnitude and duration of STING activation, Wang et al. developed dual-STING agonist micelles (D-SAM)^237^ that codeliver cGAMP and the polymer PC7A, which stabilizes STING dimers and prolongs cytosolic signaling. In MC38 tumor-bearing mice, a single intratumoral injection of D-SAM induced stronger and more durable expression of ISGs, including IFN-β, CXCL9, CXCL10, TNF-α, and IRF-7, than cGAMP or PC7A alone. These effects were observed in both tumors and tumor-draining lymph nodes. D-SAM also increased BATF3⁺ dendritic-cell infiltration and chemokine levels (CXCL10, CCL5, and IL-6), promoting memory T-cell formation. Therapeutically, D-SAM significantly suppressed tumor growth and extended median survival by up to 80% in B16F10, 4T1, and MC38 tumor models.

Finally, Chibaya et al. codelivered cGAMP and a TLR4 agonist in lipid nanoparticles combined with senescence-inducing MEK and CDK4/6 inhibitors.^238^ This synergistic strategy upregulated MHC-I and TAP1/2 in senescent tumor cells and activated DCs via both the STING and TLR4 pathways. It increased infiltration of granzyme B⁺ CD8⁺ T cells and NKp46⁺ NK cells, inhibited tumor progression, and conferred immune memory in murine pancreatic ductal adenocarcinoma models. Collectively, these studies highlight that the strategic integration of PRR agonists with stimuli-responsive nanocarriers not only enhances local antigen presentation and innate activation but also synergizes with adaptive immunity to elicit potent systemic antitumor responses, paving the way for next-generation in situ cancer vaccines (Table 3).

Son et al. first developed pathogen-mimicking sugar nanocapsules (Mann-capsules)^231^ composed entirely of microbial mannan using a template-assisted layer-by-layer (LbL) assembly technique. These hollow, redox-degradable nanostructures were designed to deliver mRNA without the need for exogenous adjuvants while mimicking PAMPs to engage innate immune receptors such as Dectin-2 and TLR4. Upon subcutaneous administration, Mann-capsules effectively activated DCs, upregulated costimulatory molecules (CD40, CD86), enhanced MHC-I–mediated antigen presentation, and promoted CD8⁺ T-cell proliferation. These findings support Mann-capsules as a modular, self-adjuvanted mRNA delivery platform capable of precise DC targeting and PRR-mediated immunoactivation, features that align with the mechanistic principles of in situ vaccination and highlight their translational potential in mRNA-based cancer immunotherapy. Building on this platform, the same team subsequently developed mannan-coated hollow polyelectrolyte nanocapsules (Mann-NCs) to amplify innate immune sensing and T-cell activation within the tumor microenvironment.^100^ Upon intratumoral injection, Mann-NCs engaged Dectin-2 and TLR4 on DCs and macrophages, triggering NF-κB–dependent transcriptional programs and inducing proinflammatory cytokines, including IL-6, TNF-α, and IL-12p70. This stimulation led to robust infiltration of CD4⁺ and CD8⁺ T cells with a Th17-skewed phenotype and a reduction in regulatory T cells (Tregs), thereby increasing the Th17/Treg ratio. Notably, even in the absence of defined antigens, the multivalent mannan surface elicited potent innate and adaptive immune activation. Coadministration with anti-OX40 antibodies further amplified T-cell responses and enhanced tumor regression, underscoring the potential of Mann-NCs as a self-adjuvanted immunomodulatory nanoplatform that bridges PRR-mediated innate sensing with adaptive antitumor immunity.

While PRR agonist delivery enhances local immune activation, the lack of efficient coordination with secondary lymphoid structures often limits the initiation of robust systemic antitumor immunity. To bridge this spatial gap, recent strategies have incorporated targeted delivery of immune-stimulatory cues to draining lymph nodes (dLNs), where antigen presentation and T-cell priming are orchestrated. Among these, engineering nanocarriers to engage the CCR7–CCL21 chemokine axis has garnered attention owing to its physiological role in directing mature DCs to lymphoid tissues. For example, nanoparticles functionalized with CCL21 or CCR7-binding peptides have demonstrated enhanced lymphatic migration and T-cell activation. A notable example is the development of an implantable alginate/collagen hydrogel encapsulating CCL21-expressing DCs (CCL21-DCs@gel),^239^ which generated sustained CCL21 gradients, promoted immune cell infiltration, and significantly potentiated radiotherapy (RT) by suppressing both primary tumor growth and distant metastasis in vivo.

However, this chemotactic axis is also known to be hijacked by tumor cells to facilitate lymph node metastasis. Clinical analyses have revealed that metastatic tumor cells frequently overexpress CCR7 and exploit endogenous CCL19/CCL21 gradients to migrate into lymphatic tissues. These metastatic niches not only show higher CCR7 levels but also elevated expression of inhibitory checkpoints such as PD-1, LAG-3, and TIM-32. To counteract this, a CCR7 antagonist peptide (TC6-D3) was developed,^240^ which effectively blocked CCR7–ligand interactions, inhibited ERK1/2-mediated tumor migration, and restored CD8⁺ T-cell cytotoxicity in both primary tumors and lymph nodes. These dual insights underscore the context-dependent role of CCR7–CCL21 signaling, suggesting that its therapeutic exploitation must be carefully modulated to promote APC migration and immune activation while minimizing the risk of tumor dissemination.

### Non-nanomedicine strategies

In addition to nanomedicine-based platforms, we also reviewed several non-nanomedicine strategies, including radiotherapy (RT), photodynamic therapy (PDT), and hydrogel-based sustained-release systems.

Radiotherapy primarily eliminates tumor cells through ROS-mediated DNA damage and, to a lesser extent, by disrupting cellular structures such as the plasma membrane. In addition to its cytotoxic effects, RT also triggers ICD and promotes the release of a broad spectrum of tumor antigens, thereby facilitating dendritic-cell activation and cytotoxic T-cell priming.

Demaria et al. demonstrated that fractionated RT significantly increased intratumoral CD8⁺ T-cell infiltration and synergized with PD-1/PD-L1 blockade to enhance tumor regression and survival.^241^ Similarly, Dovedi et al. investigated whether fractionated RT combined with PD-1 blockade could improve post-tumor challenge survival.^242^ In mice bearing CT26 colon carcinoma, 4T1 triple-negative breast cancer, or B16 melanoma, the combination of fractionated RT and anti–PD-1/PD-L1 therapy markedly enhanced tumor control and overall survival compared with either monotherapy. Most treated animals achieved long-term survival,66–80% of treated animals survived beyond 100 days, and were resistant to tumor rechallenge. RT rapidly increased PD-1 expression on tumor-infiltrating CD8⁺ T cells, suggesting that local RT elicits potent CD8⁺ T-cell responses that are constrained by PD-1/PD-L1 signaling. These findings underscore the importance of early PD-1 pathway blockade for establishing durable antitumor immunity.

Photodynamic therapy (PDT) induces localized oxidative stress that promotes tumor cell death and the release of antigens and DAMPs, thereby enhancing DC maturation and cross-priming of T cells. Mechanistically, PDT triggers ROS-dependent apoptosis, necrosis, or autophagy, disrupts the tumor vasculature, leading to hypoxia-induced infarction, and generates inflammatory signals that propagate systemic antitumor immunity.^243^ Recent studies have shown that combining PDT with immunotherapeutic interventions can markedly potentiate these effects. Hwang et al. demonstrated that combining PDT with a TLR5 agonist-based tumor-specific peptide vaccine (FlaB-Vax), followed by PD-1 blockade, elicited potent systemic and local antitumor immunity in bilateral B16F10 melanoma models.^244^ This combination markedly enhanced tumor-infiltrating effector memory CD8⁺ T cells and systemic IFN-γ production, indicating strong cytotoxic T-cell responses. It also increased CD103⁺ dendritic cells and CXCL9/10 secretion, promoting CXCR3-dependent T-cell recruitment. PD-1 blockade further enhanced efficacy by sustaining CD8⁺ T-cell activation and overcoming adaptive immunosuppression, resulting in complete tumor regression.

Similarly, Zhang et al. developed a biomimetic nanoemulsion (PHD@PM) camouflaged with PD-1-expressing HEK293T cell membranes to codeliver photosensitizers and immune checkpoint proteins for synergistic photodynamic immunotherapy.^245^ The core nanoemulsion comprised sinoporphyrin sodium (DVDMS)-bound human serum albumin (HSA) and the oxygen carrier perfluorotributylamine (PFTBA), enabling oxygen supply and light-triggered ROS generation within hypoxic tumors. In 4T1 breast tumors, two laser treatments eradicated primary tumors and achieved long-term survival, whereas controls showed only partial inhibition. PHD@PM alone increased mature DCs from 20.1% to 32.6% and further to 40.1% upon laser irradiation, confirming synergistic activation via PD-1-mediated immune modulation and PDT-induced ICD. In bilateral tumor models, PHD@PM with a laser also prevented metastasis, showing efficacy comparable to that of anti-PD-L1 therapy. Collectively, this biomimetic PD-1 membrane-coated nanoemulsion combined oxygenated PDT and immune checkpoint engagement to achieve potent primary and abscopal tumor control. Despite these promising results, the clinical translation of PDT remains limited by shallow tissue penetration of visible light, inconsistent ROS generation in deep-seated tumors, and photosensitivity-related adverse effects.^246^

Finally, hydrogel-based systems provide sustained local release of cytokines or immunomodulators while reducing systemic exposure.^247^ Ji et al. developed an implantable biopolymer hydrogel coloaded with the TLR7/8 agonist resiquimod (R848) and an anti-OX40 antibody (aOX40) to achieve sequential activation of innate and adaptive immunity for postoperative colorectal cancer therapy.^248^ In CT26 colon carcinoma-bearing BALB/c mice, the hydrogel was implanted postsurgery to mimic residual disease. Sequential release of R848 and aOX40 activated NK and dendritic cells, induced IFN-γ production, and promoted CD4⁺/CD8⁺ T-cell infiltration. This combination hydrogel (BI(R848 + aOX40)) achieved complete tumor eradication and long-term recurrence-free survival, outperforming single-agent or soluble controls. In bilateral CT26 models, BI(R848 + aOX40) suppressed the growth of untreated distant tumors by 83%, demonstrating strong systemic immunity. Collectively, this localized immune implant effectively prevented postoperative recurrence and distant metastasis by orchestrating temporally resolved innate and adaptive immune activation while minimizing systemic toxicity. However, these systems still face challenges such as uncontrolled degradation rates and localized inflammation.^249^

Taken together, these non-nanomedicine strategies demonstrate significant potential in reshaping the tumor immune microenvironment and enhancing antitumor immunity. Nonetheless, their less precise targeting, inconsistent biodistribution, and dose-limiting toxicities underscore the unique advantages of nanomedicine platforms, such as precise tumor localization, multifunctional codelivery, and tunable pharmacokinetics, in advancing combinatorial in situ cancer vaccination.

## Clinical translation

The clinical translation of in situ cancer vaccines has advanced rapidly in the past decade, driven by their ability to leverage the full antigenic repertoire of tumors and induce systemic antitumor immunity. Early-phase clinical studies have demonstrated that intratumoral administration of immunoadjuvants can convert “cold” tumors into “hot” immunogenic lesions, leading to durable systemic responses in subsets of patients. Among the most extensively studied in situ cancer vaccines are Toll-like receptor 9 (TLR9) agonists, such as SD-101^250^ and vidutolimod (CMP-001).^251^ SD-101 is a synthetic CpG oligonucleotide that activates plasmacytoid dendritic cells (pDCs) through TLR9, promoting robust type I interferon secretion, enhanced antigen presentation, and the priming of cytotoxic CD8⁺ T cells. Preclinical studies in mice have shown that intratumoral injection of SD-101 combined with systemic anti–PD-1 therapy can induce complete and durable rejection of nearly all injected tumors as well as most distant, noninjected tumors.^252^ Clinically, in a study involving 27 patients with low-grade non-Hodgkin lymphoma, direct injection of SD-101 into tumors combined with low-dose radiation therapy not only activated local immune responses but also induced systemic abscopal effects.^253^

Similarly, STING agonists (e.g., ADU-S100, MK-1454) and dsRNA mimetics (e.g., BO-112) are under active clinical investigation. As a monotherapy, MIW815 (ADU-S100)^254^ was well tolerated in patients with advanced solid tumors and lymphomas, with no maximum tolerated dose reached. Although clinical efficacy was limited, 94% of injected lesions were stable or reduced in size, accompanied by increased systemic inflammatory cytokines and peripheral T-cell clonal expansion. In patients with anti–PD-1-resistant advanced melanoma, intratumoral injection of BO-112 combined with intravenous pembrolizumab achieved an objective response rate of 25%, including a complete response rate of 10% and a partial response rate of 15%, with stable disease observed in 40% of patients. The 24-month survival rate reached 54%, and treatment-related grade 3−5 adverse events occurred in only 9.5% of patients, indicating a favorable safety profile.^255^

The ongoing phase I/II clinical trial (NCT03789097) is investigating an in situ cancer vaccine strategy combining intratumoral Flt3L, local radiation, and the TLR3 agonist poly-ICLC with systemic pembrolizumab in patients with indolent non-Hodgkin lymphoma (iNHL), metastatic breast cancer (MBC), and head and neck squamous cell carcinoma (HNSCC). Interim data demonstrated that the regimen was generally well tolerated, with most treatment-related adverse events limited to mild injection-site reactions and flu-like symptoms, and only two grade 3 events were reported (self-limiting fever and pembrolizumab-associated colitis). Importantly, early signals of systemic antitumor activity were observed, including one complete response, two partial responses, and one case of stable disease, while several patients showed metabolic reduction of tumor burden. Notably, one heavily pretreated estrogen receptor-positive and progesterone receptor-positive (ER/PR-positive) MBC patient achieved a durable partial response with no new lesions for at least six months.^256^ In light of these clinical findings, Table 4 provides an overview of representative in situ cancer vaccine trials, summarizing their cancer types, stages of clinical development, and limitations.

**Table 4:** Summary of key clinical trials of in situ cancer vaccines

Strategy/Agent	Trial ID/NCT No.	Cancer type	Strategy status	Notes/Limitations
Intratumoral SD-101 (TLR9 agonist) + low-dose radiation	Phase I/II	Low-grade non-Hodgkin lymphoma (NHL)	Activated local immune responses; observed systemic abscopal effects	Optimization of dosing and injection reproducibility needed
MIW815 (ADU-S100, STING agonist)	Phase I [NCT02675439]	Advanced solid tumors and lymphomas	Well tolerated; 94% of injected lesions stable/reduced; systemic cytokine induction; T-cell clonal expansion	Limited clinical efficacy; further combination strategies required
BO-112 (dsRNA mimetic) + pembrolizumab	Phase II [NCT04508140]	Anti–PD-1-resistant advanced melanoma	ORR 25% (CR 10%, PR 15%); SD 40%; 24-month survival 54%; grade 3–5 AEs 9.5%	Need for broader validation and longer follow-up
Flt3L + local radiation + poly-ICLC (TLR3 agonist) + pembrolizumab	Phase I/II [NCT03789097]	iNHL, metastatic breast cancer (MBC), head and neck squamous cell carcinoma (HNSCC)	Generally, well tolerated; mild flu-like symptoms; early systemic responses, including CR and durable PR	Long-term efficacy data pending; optimization of regimen scheduling
Nanomedicine-based formulations (various agents)	Preclinical to early-phase clinical	Multiple tumor types	Enhanced delivery and immunogenicity demonstrated in models	Regulatory and manufacturing challenges for complex formulations cancers

Despite these encouraging advances, several translational and practical barriers still limit the widespread clinical application of in situ cancer vaccines. One major challenge lies in the standardization of intratumoral (IT) administration techniques. Parameters such as needle gauge, injection rate, number of passes, and imaging guidance vary substantially across clinical centers, resulting in inconsistent drug distribution and retention.^257,258^ These procedural differences are particularly impactful in deep or poorly accessible lesions such as the liver, lung, or pancreas, which often require ultrasound, CT, or endoscopy-guided injections.

Moreover, maintaining local depot residence and achieving uniform antigen exposure across multiple lesions are crucial for robust innate pathway activation. Emerging delivery technologies, such as injectable hydrogels, in situ gelling polymers, and stimuli-responsive nanoparticles, can prolong retention and control drug release. Developing an injectable, in situ gelling hydrogel requires precise control over rheological properties, cross-linking mechanisms, and degradation rates to ensure injectability, mechanical stability, and predictable release kinetics within the tumor microenvironment. For instance, alginate CpG hydrogels or chitosan-based depots have demonstrated sustained DC activation and prolonged immune stimulation in preclinical melanoma models, yet such materials also introduce workflow complexity and manufacturing burden.^259^ In particular, ensuring batch-to-batch consistency for critical quality attributes (CQAs), such as polymer molecular weight, gelation kinetics, and drug encapsulation efficiency, significantly escalates both cost and regulatory complexity.

Safety and toxicity management also require careful optimization. Local inflammation, ulceration, or injection-site necrosis must be balanced against sufficient innate activation. One potential risk is the induction of nonspecific immune responses against antigens that are also expressed on normal tissues, since many tumor-associated antigens are not strictly tumor-specific. Such cross-reactivity could inadvertently damage healthy cells. Another concern is the possibility of autoimmune reactions, in which heightened immune activation may break immune tolerance and trigger systemic or organ-specific autoimmunity. For example, intra-articular injection of CpG-ODNs in animal models has been shown to induce arthritis in vivo.^260^ In addition, the use of potent ICD inducers such as chemotherapeutics, phototherapies, or oncolytic agents carries the risk of collateral killing of healthy, nonmalignant cells within or near the injection site. These adverse effects not only compromise patient safety but may also limit therapeutic efficacy by inducing excessive inflammation or tissue injury.^261^

When IT therapies are combined with ICIs, systemic immune-related adverse events (irAEs), such as hepatitis, pneumonitis, or thyroiditis, may occur, highlighting the need for adaptive dosing, step-up schedules, and close clinical monitoring. In clinical testing, ADU-S100 + anti-PD-1 combinations produced manageable grade 1–2 injection-site pain and flu-like symptoms but limited abscopal effects (NCT03937141).^262^ Similarly, CMP-001 + nivolumab yielded local tumor regression and occasional systemic responses but frequent grade 2 inflammation at injection sites (NCT02680184).^263^ These experiences emphasize that dosing frequency, lesion selection, and combination sequence critically influence tolerability and systemic benefit.

Clinical trial design and biomarker integration represent another frontier. Future studies should prespecify injected versus noninjected lesions, incorporate imaging criteria that account for pseudo-progression or mixed responses, and include pharmacodynamic endpoints confirming on-mechanism activation within the tumor, such as type I IFN signatures, dendritic-cell maturation markers (CD40, IL-12), and chemokine induction (CXCL9/10). Peripheral immune monitoring (TCR repertoire breadth, cytokine panels) can delineate systemic immune expansion. Patient selection criteria should consider tumor accessibility, baseline myeloid composition, and T-cell inflamed gene expression profiles, while low TMB cancers such as pancreatic adenocarcinoma or prostate cancer may particularly benefit from ICD adjuvant combinations that enhance antigen diversity and cross-presentation. For instance, a pilot clinical trial using locally sustained-release LSAM-PTX (NCT03077685) in patients with advanced pancreatic cancer demonstrated that intratumoral administration was feasible and well tolerated, promoting local immune infiltration and inflammatory cytokine induction without systemic toxicity.^264^ Integrating predictive biomarkers for responders versus nonresponders and systematically reporting both positive and negative outcomes will accelerate rational clinical translation.

Collectively, while in situ cancer vaccines have established a strong scientific and clinical foundation, ongoing refinement in delivery, trial design, and biomarker integration will be essential to achieve broader regulatory approval and integration into standard-of-care regimens.

## Future perspectives and challenges

In situ cancer vaccination represents a transformative immunotherapeutic strategy that leverages the tumor itself as a source of antigens and immune stimulation, bypassing the need for predefined antigen identification and enabling broad immune responses against tumor-associated and neoantigens. Despite these advantages, several scientific, technical, and translational challenges must be addressed to facilitate its broad clinical application. Reliable induction of ICD across diverse tumor types remains a major hurdle. While conventional modalities such as chemotherapy, radiotherapy, and photodynamic therapy can promote ICD, their immunogenicity is often context-dependent and may be insufficient in tumors with apoptotic resistance or profound immune tolerance. Emerging evidence suggests that targeting nonapoptotic regulated cell death pathways, including necroptosis, pyroptosis, and ferroptosis, could elicit stronger innate and adaptive immune responses, but strategies to precisely and safely induce these pathways in situ remain under active investigation.

The immunosuppressive TME, characterized by hypoxia, aberrant vasculature, high interstitial pressure, and suppressive immune cell populations, further impedes effective in situ cancer vaccination. Nanotechnology offers promising solutions by enabling the codelivery of ICD inducers, immunostimulatory agents, and TME modulators with spatial and temporal precision. However, further efforts are required to optimize nanocarrier design, improve tumor targeting, and ensure safety, scalability, and clinical manufacturability. Similarly, robust activation of innate immune pathways is critical for dendritic-cell maturation and T-cell priming. Although synthetic agonists of PRRs, such as TLR and STING agonists, have shown strong potential, their effective delivery into poorly perfused or fibrotic tumors remains challenging, and systemic administration carries a risk of off-target inflammation. Tumor-targeted or stimuli-responsive delivery platforms will be essential for improving safety and maximizing efficacy.

Finally, integration with complementary immunotherapies, such as immune checkpoint inhibitors or adoptive cell therapies, may be essential to achieving durable responses in immunologically “cold” tumors. Rational combination strategies and optimal treatment schedules will require continued investigation in both preclinical models and clinical trials.

### Harnessing DAMP/PAMP signaling to improve in situ cancer immunotherapy

Beyond addressing these barriers, harnessing the coordinated interplay between DAMPs and PAMPs offers a promising strategy for next-generation in situ vaccination.^76^ These two immunostimulatory classes activate distinct yet complementary innate signaling pathways. While DAMPs signal cellular stress or damage through receptors such as TLR4, cGAS–STING, and NLRs, PAMPs mimic microbial infection and trigger proinflammatory responses via PRRs, particularly TLRs^165^ and C-type lectin receptors (CLRs). While either class of stimuli can independently drive antitumor immunity, the simultaneous activation of these two signaling pathways enhances the cross-priming ability of DCs, boosts CD8⁺ T-cell responses, and ultimately drives more robust systemic antitumor immunity.^1,265^

Despite this reasoning, there are still many challenges. Some PAMPs may indirectly trigger ICDs themselves, making it difficult to distinguish primary adjuvant effects from secondary immunogenic damage.^7,266^ Additional complexity arises from the immunosuppressive tumor microenvironment and potential systemic toxicities associated with high-dose PAMPs.^267^ Recent research has advanced the design of nanoparticle- and hydrogel-based codelivery systems that enable the spatiotemporal synchronization of DAMP and PAMP signals. These delivery platforms offer precise control over release kinetics, enable site-specific immune activation within the TME, and reduce systemic exposure and off-tumor, on-target toxicity. For example, injectable hydrogel-based nanoparticles have been developed to deliver doxorubicin, a chemotherapeutic known to induce ICD in combination with TLR agonists such as CpG oligodeoxynucleotides. These formulations enhance the local release of tumor antigens and DAMPs while simultaneously stimulating DC maturation via PRR engagement. Similarly, combining DAMP-inducing adjuvants such as 2-hydroxypropyl-β-cyclodextrin (HP-β-CyD) with PAMP adjuvants such as K3 CpG oligodeoxynucleotide (a TLR9 agonist) has been shown to elicit synergistic immune responses by concurrently activating type-2 and type-1 immunity. In a mouse model of influenza split vaccination, this combination not only enhanced antigen-specific IgG1 and IgG2c responses but also promoted IL-4 and IFN-γ production while suppressing IgE induction, thereby improving both efficacy and safety profiles. The dual adjuvant formulation significantly improved protection against lethal viral challenge, even at high viral loads, highlighting the potential of parallel DAMP/PAMP activation to augment vaccine immunogenicity and durability.^268^

In summary, in situ cancer vaccination represents a compelling immunotherapeutic paradigm with the potential to harness tumor heterogeneity and elicit durable systemic immunity. Ongoing advances in nanomedicine, ICD induction, and immune modulation will be critical to overcoming current limitations and translating this strategy into effective, broadly applicable clinical interventions.
